# Oligosaccharide Binding Proteins from *Bifidobacterium longum* subsp. *infantis* Reveal a Preference for Host Glycans

**DOI:** 10.1371/journal.pone.0017315

**Published:** 2011-03-15

**Authors:** Daniel Garrido, Jae Han Kim, J. Bruce German, Helen E. Raybould, David A. Mills

**Affiliations:** 1 Department of Food Science and Technology, University of California Davis, Davis, California, United States of America; 2 Foods for Health Institute, University of California Davis, Davis, California, United States of America; 3 Functional Glycobiology Program, University of California Davis, Davis, California, United States of America; 4 Robert Mondavi Institute for Wine and Food Sciences, University of California Davis, Davis, California, United States of America; 5 Department of Viticulture and Enology, University of California Davis, Davis, California, United States of America; 6 Department of Anatomy, Physiology and Cell Biology, University of California Davis, Davis, California, United States of America; University of South Florida College of Medicine, United States of America

## Abstract

*Bifidobacterium longum* subsp. *infantis (B. infantis)* is a common member of the infant intestinal microbiota, and it has been characterized by its foraging capacity for human milk oligosaccharides (HMO). Its genome sequence revealed an overabundance of the Family 1 of solute binding proteins (F1SBPs), part of ABC transporters and associated with the import of oligosaccharides. In this study we have used the Mammalian Glycan Array to determine the specific affinities of these proteins. This was correlated with binding protein expression induced by different prebiotics including HMO. Half of the F1SBPs in *B. infantis* were determined to bind mammalian oligosaccharides. Their affinities included different blood group structures and mucin oligosaccharides. Related to HMO, other proteins were specific for oligomers of lacto-N-biose (LNB) and polylactosamines with different degrees of fucosylation. Growth on HMO induced the expression of specific binding proteins that import HMO isomers, but also bind blood group and mucin oligosaccharides, suggesting coregulated transport mechanisms. The prebiotic inulin induced other family 1 binding proteins with affinity for intestinal glycans. Most of the host glycan F1SBPs in *B. infantis* do not have homologs in other bifidobacteria. Finally, some of these proteins were found to be adherent to intestinal epithelial cells *in vitro*. In conclusion, this study represents further evidence for the particular adaptations of *B. infantis* to the infant gut environment, and helps to understand the molecular mechanisms involved in this process.

## Introduction

The distal portion of the human gastrointestinal tract is colonized by an impressive number of microorganisms, which are collectively referred as the intestinal microbiota. The environment they occupy is considered to be rich in complex dietary and host-derived oligosaccharides, which serve as an important carbon source for many of these microbes. In general, members of the intestinal microbiota devote an important percentage of their genomes to the utilization of complex carbohydrates [1], [2]. This specialization requires specific intra or extracellular glycosyl hydrolases to break down the complex oligosaccharides arriving to the colon, and also transport mechanisms to import intact or processed glycans inside the bacterial cell. Bacterial ABC transporters involved in the import of oligosaccharides show an exquisite specificity, conferred by Solute Binding Proteins (SBPs) [3].

*Bifidobacterium* species represent an important percentage of the total colonic microbiota in infants [4], and they remain at significant levels in adults [5]. In general they are considered beneficial microorganisms. Some strains in this genus are noteworthy as probiotics, and they represent one of the main targets in prebiotic interventions [6]. In particular, bifidobacteria are efficient in the utilization of complex oligosaccharides, such as fructo-oligosaccharides (FOS) [7], galacto-oligosaccharides (GOS) [8] and inulin [9].

In the last decades several studies have associated breast-feeding with higher concentrations of bifidobacteria in infant feces [10], [11]. Formula fed infants seem to have lower numbers of *Bifidobacterium*, even though it has been argued that improvements in the composition of infant formulas hsve reduced this difference [12]. Formula fed infants are also characterized by the presence of species commonly found in adults, such as *Bifidobacterium adolescentis* and *Bifidobacterium pseudocatenulatum*, while breast-fed infants are colonized more often with *Bifidobacterium breve* and *B. infantis* [13]. *Bifidobacterium bifidum* and *Bifidobacterium longum* subsp. *longum* (*B. longum*), can be found both in infants and adults [14].

Human milk oligosaccharides (HMO) are abundant in breast milk and they are thought to act as prebiotics selecting for specific bacterial populations in the infant gut. They are characterized by a lactose molecule at the reducing end to which subunits of lacto-N-biose (LNB; type 1 chain; Galβ1–3GlcNAc) or N-acetyl-lactosamine (type 2 chain; Galβ1–4GlcNAc) are attached in tandem [15]. Fucose and sialic acid residues can be located at terminal positions. 200 different HMO structures have been determined, however, four molecular masses can represent up to the 70% of the total molecules, including isomers of lacto-N-tetraose (Galβ1–3GlcNAcβ1–3Galβ1–4Glc; LNT), lacto-N-neotetraose (Galβ1–4GlcNAcβ1–3Galβ1–4Glc; LNnT), lacto-n-hexaose (LNH), monofucosyl-lacto-N-hexaose and difucosyl lacto-N-hexaose [16]. Interestingly, HMO structures resemble the lacto and neolacto family of glycolipids [17], and polylactosamine chains are characteristic in glycoproteins [18]. Mucin oligosaccharides also share structural similarity with HMO [19].

Recently *B. longum*, *B. infantis, B. breve* and *B. bifidum* were characterized by their capabilities for consuming HMO [20]. Particularly, a gene cluster for metabolism and import of LNB and galacto-N-biose (Galβ1–3GalNAc; GNB), a core substructure in mucin glycans, is found in these microorganisms [21]. Also enzymes involved in the deconstruction of HMO and the metabolism of its constituents have been characterized [22], [23]. Different strategies exist for HMO consumption. *B. longum*, *B. breve* and *B. bifidum* seem to prefer LNT [24], and potentially rely on extracellular glycosyl hydrolases for reduction of more complex HMO as well as mucin glycans [25], while *B. infantis* apparently has expanded its metabolic capabilities to use a higher variety of HMO molecules, specializing in the import of complex carbohydrates and intracellular degradation [26]. The genome of this bacterium has been recently sequenced [27], and several features indicated a specialization in carbohydrate utilization. In addition to the LNB/GNB cluster, *B. infantis* possesses two additional gene clusters containing ABC transporters and glycosyl hydrolases predicted to be involved in HMO utilization. These clusters were also found to contain an important number of Family 1 Solute Binding Proteins (F1SBPs), predicted to import oligosaccharides associated to ABC transporters.

Genomic analysis suggests that *B. infantis* evolved from a plant-derived glycan utilization genotype, to be competitive in the infant colon [27]. Interestingly, all available carbon sources in this environment are oligosaccharides from human origin, including a significant concentration of HMO arriving undigested to the distal colon, as well as intestinal secretions and glycoconjugates from epithelial cells. The F1SBPs in *B. infantis* might be important to understand the adaptations of this microorganism to the infant gut, as well as the range of oligosaccharides it is able to import into the cell and use as a carbon source. In this work we have characterized *B. infantis* F1SBPs oligosaccharide affinities, expression during growth on different prebiotics and interactions with epithelial cells in order to better understand the complex nature for oligosaccharide foraging by this important intestinal species.

## Results

### Several *B. infantis* F1SBPs bind host glycans

**Figure 1:**
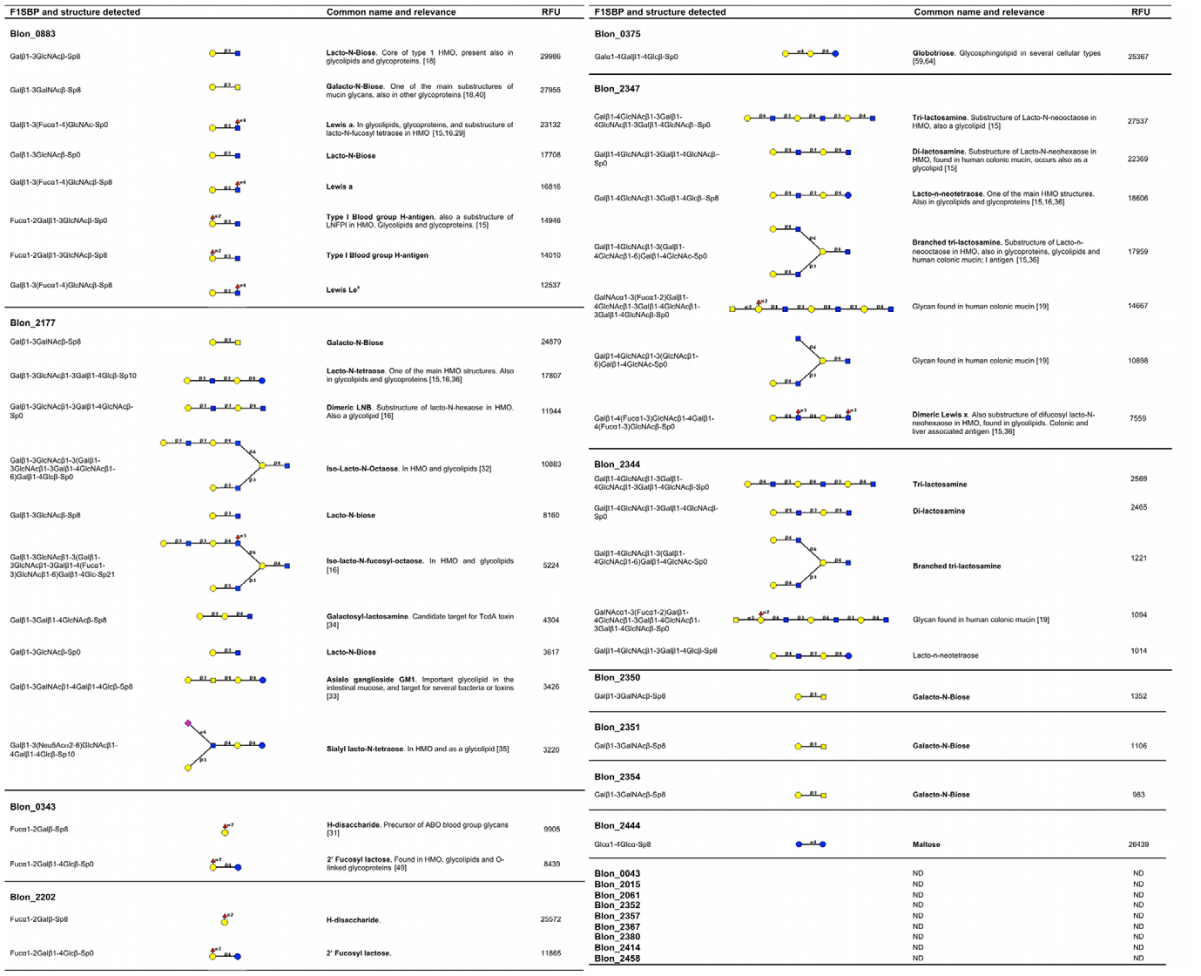
Oligosaccharides recognized by *B. infantis* F1SBPs in the Glycan Array v3.1. Oligosaccharides are attached to the array using different spacer arms. Sp0: -CH2CH2NH2; Sp8: -CH2CH2CH2NH2; Sp10: -NHCOCH2NH; Sp21: -N(CH3)-O-(CH2)2-NH2. RFU: Relative Fluorescence Units. Yellow circles, galactose; blue circles, glucose; yellow squares, N-Acetyl-galactosamine; blue squares, N-Acetyl-glucosamine; red triangles, fucose; pink diamonds, sialic acid. ND: Not detected.

The genome of *B. infantis* ATCC 15697 has twenty genes containing the Pfam motif *Sbp_bac_1*, Family 1 of Solute Binding Proteins (F1SBPs), with predicted affinity for oligosaccharides [3]. Their abundance in this genome is as much as twice compared to other bifidobacterial genomes (Table 1). The F1SBPs genes from *B. infantis* ATCC 15697 were PCR amplified, removing the lipoprotein anchor sequences, and then cloned, expressed in *Escherichia coli* and purified as glutathione-S-transferase (GST) fusion proteins. The oligosaccharide binding properties of the recombinant F1SBPs were analyzed using the Mammalian Glycan Array v3.1 [28], provided by the Consortium for Functional Glycomics. This version of the array contains more than 400 different glycans mainly present in mammalian cells. Eleven F1SBPs from *B. infantis* bound at least one carbohydrate in this array, suggesting a broad nutritional preference for host-derived glycans. Figure 1 catalogs the glycans detected by these proteins and their relative affinities (Table S2 list the oligosaccharides present in the array).

**Table 1:** Representation of the Pfam01547 motif in bifidobacterial genomes.

Sequenced genome		Pfam01547 hits	
*Bifidobacterium adolescentis* ATCC 15703		9	
*Bifidobacterium animalis lactis* AD011		6	
*Bifidobacterium animalis* subsp. *lactis* BB-12		5	
Bifidobacterium animalis subsp. *lactis* Bl-04	6		
*Bifidobacterium bifidum* NCIMB 41171 (Draft)	3		
*Bifidobacterium bifidum* PRL2010	3		
*Bifidobacterium breve* DSM 20213 (Draft)	16		
*Bifidobacterium catenulatum* DSM 16992 (Draft)	11		
*Bifidobacterium dentium* ATCC 27678	23		
*Bifidobacterium dentium* Bd1	23		
*Bifidobacterium longum* DJO10A	16		
*Bifidobacterium longum* NCC2705	11		
*Bifidobacterium longum infantis* ATCC 15697	20		
*Bifidobacterium pseudocatenulatum* DSM 20438 (Draft)	15		

The specificities of some F1SBPs narrowed to relatively few substrates. For example, Blon_0375 bound solely to the trisaccharide globotriose (Galα1–4Galβ1–4Glc), while Blon_2444 was found to have affinity for maltose. Blon_0343 and Blon_2202 share significant homology [27], and they both recognized two related oligosaccharides: Fucα1–2Gal, the precursor for the ABO blood group, and Fucα1–2Galβ1–4Glc, 2′ Fucosyl lactose (2FL). These structures occur as epitopes in more complex glycans and many were present in the array (Table S2), however these proteins only recognized their free forms.

Other F1SBPs showed a broader and more flexible range of glycans recognized in the array. Blon_0883 and Blon_2177 bound type 1 glycans, characterized by the disaccharide Galβ1–3GlcNAc (LNB), as well as Galβ1–3GalNAc (GNB), one of the core substructures in mucin oligosaccharides. Interestingly, Blon_0883 also strongly bound to Lewis a and the type 1 H-trisaccharide, two main epitopes present in glycolipids and glycoproteins in several cell types including the intestinal epithelium [29], [30], [31]. However, similarly to Blon_0343 and Blon_2202, Blon_0883 did not recognize these structures as part of more complex oligosaccharides. Blon_2177 also bound other type 1 polymers, such as LNT, LNH, lacto-N-octaose (LNO), all abundant structures in HMO [32], as well as other mammalian carbohydrates such as asialo GM1 [33], the putative receptor for the *Clostridium difficile* toxin TcdA [34], and sialyl-LNT [35], albeit the latter structures were bound at a lower affinity (Figure 1).

*B. infantis* possesses a unique cluster of ABC transporters and glycosyl hydrolases that was predicted to be important in the HMO+ phenotype of this bacterium. This cluster contains 6 F1SBPs closely related at the sequence level [27]. Blon_2344 and Blon_2347 specifically recognized type 2 glycans (Figure 1), binding with different affinities linear and branched polylactosamines, as well as lacto-n-neotetraose (LNnT) and difucosyl lacto-N-hexaose. These structures are characteristic in HMO as well as in glycolipids and glycoproteins [36]. These two F1SBPs also presented affinity for complex neutral oligosaccharides found in human colonic mucins [19] and thus are candidates for interaction with epithelial surfaces and mucins. In the same gene cluster, Blon_2350, Blon_2351 and Blon_2354 specifically detected GNB, supporting their role in binding and/or import of mucin oligosaccharides. However, the low affinity of these latter F1SBPs suggests that they may prefer other glycans not present on the array.

### F1SBP expression during growth on HMO

If F1SBPs are involved in binding and transport of complex oligosaccharides to be used as a carbon and energy source *in situ*, one would predict that their expression would be induced by the presence of such glycans. To examine this, *B. infantis* cells were grown on lactose, HMO, inulin, fructooligosaccharides (FOS) and galactooligosccharides (GOS) as the only carbon sources, and the relative expression level for each F1SBP was studied using qPCR. The presence of these proteins inside the cells was also determined using a proteomic approach.

**Figure 2:**
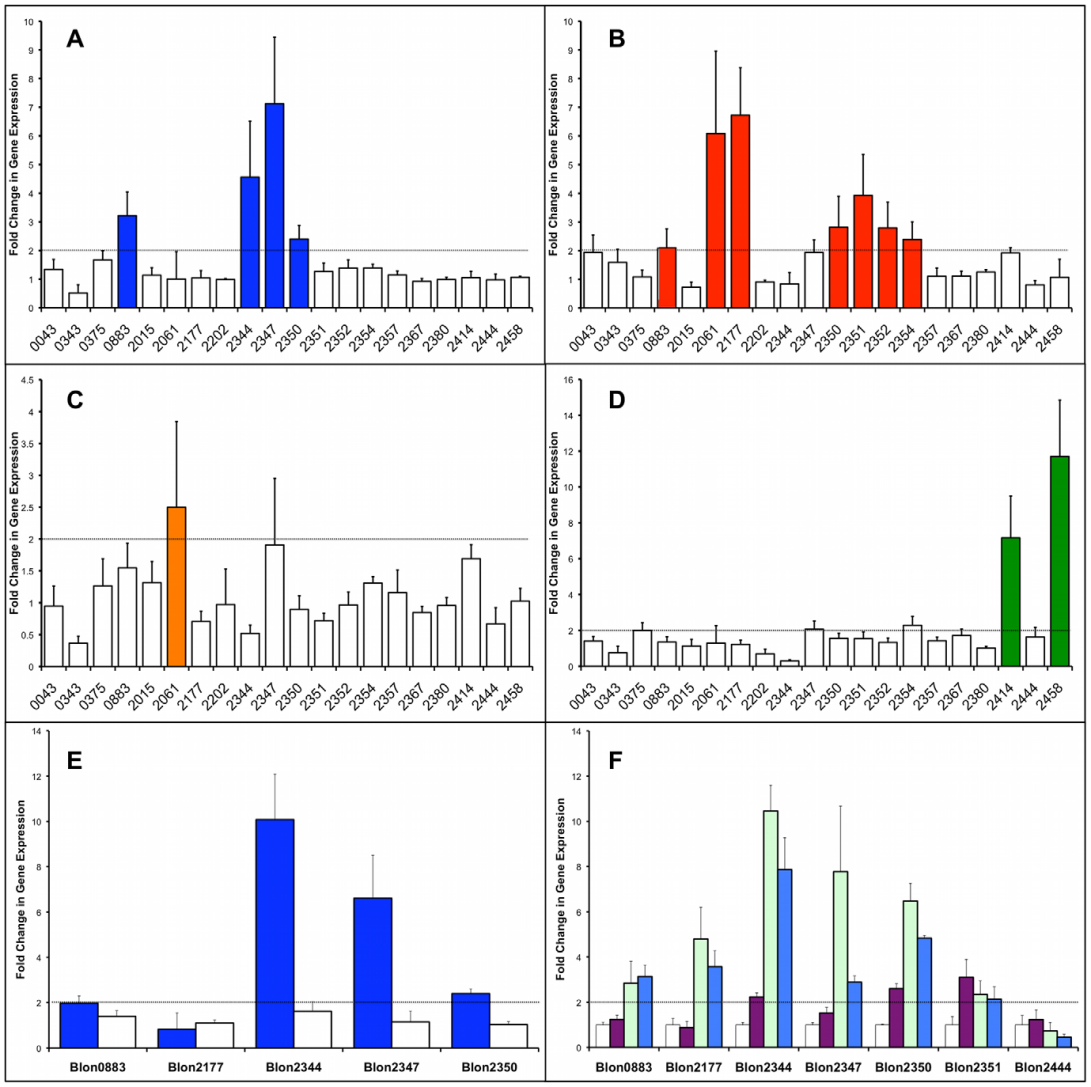
Gene expression of *B. infantis* F1SBPs growing under different prebiotics as the sole carbon source. Numbers in the x-axis represent each F1SBP locus tag (Blon_). A: 2% HMO; B: 2% Inulin; C: 2% FOS; D: 2% GOS. Colored bars indicated F1SBPs with a change in gene expression higher than two fold; E: relative expression levels for some HMO-induced genes in exponential (blue bars) and stationary phase (white bars); F: relative expression levels for some F1SBPs under growth with GNB (purple bars), LNT (green bars) and LNnT (blue bars) as the only carbon source. Levels for growth on lactose (white bars) are shown as a reference.

Growth on HMO as the only carbon source induced the expression of four F1SBP genes in the exponential phase, Blon_0883, Blon_2344, Blon_2347 and Blon_2350 (“induced” was defined as a minimum two-fold increase in gene expression; Figure 2A). Proteomics also confirmed the expression of these proteins (Table 2). These F1SBPs were not expressed in stationary phase (Figure 2E). Given the affinities of these F1SBPs for HMO structures, the expression results suggest that HMO induces F1SBPs involved in its own import. Moreover, the predominant HMO species consumed by *B. infantis* are consistent with the glycans recognized by F1SBPs expressed during growth on HMO, such as LNnT, LNnH and mono and difucosyl LNH [24]. Since these four F1SBPs also recognized other oligosaccharides present in mucins or glycoconjugates, the presence of HMO likely induces the import of mucin and epithelial glycans and/or facilitates adhesion of *B. infantis* to mucins or epithelial surfaces.

**Table 2:** Proteomic analysis of F1SBPs in *B. infantis* cells growing under different substrates.

Locus tag (Blon_)^a^	GOS				FOS				Inulin				HMO				
	log(e)^b^	P_uniq_ c	SpC_T_ d	%^e^	log(e)	P_uniq_	SpC_T_	%	log(e)	P_uniq_	SpC_T_	%		log(e)	P_uniq_	SpC_T_	%
0883									−70.7	7	10	25		−21.6	3	3	13
2061					−135.2	13	54	60	−193	18	131	75		−3.2	1	1	7
2177									−61	7	7	31					
2344														−63.9	7	15	29
2347														−60.5	4	12	17
2350														−29.5	1	1	6
2351									−13	2	3	9					
2354									−4	1	3	6					
2414	−27.1	3	4	14													

a Only F1SBPs found in the proteome of *B. infantis* are shown.

b Log(e): protein probability BY X! Tandem.

c P_uniq_: a number of unique peptides used for the identification of a protein. Only the peptides with ≥−2 were used.

d SpC_T_: a number of MS/MS spectra assigned for the identification of a protein.

e %: percentile of sequence coverage. Listed proteins have ≥95% of the protein probability by protein prophet algorithm when the peptides with ≥95% of probability (by peptide prophet algorithm) were used.

The relative gene expression for a number of HMO and mucin binding F1SBPs was then determined using LNT and lacto-N-neotetraose (LNnT), representative structures of type 1 and type 2 HMO respectively, and GNB as the only available carbon sources. All four F1SBPs induced by HMO (Blon_0883, Blon_2344, Blon_2347 and Blon_2350) showed an increase in their expression during growth on LNT as well as LNnT (Figure 2F) regardless of their own affinity for type 1 or type 2 chains as determined in the glycan array. This indicates that the induction of proteins involved in HMO import does not depend on the isomeric differences between Galβ1–3GlcNAc and Galβ1–4GlcNAc in HMO.

**Figure 3:**
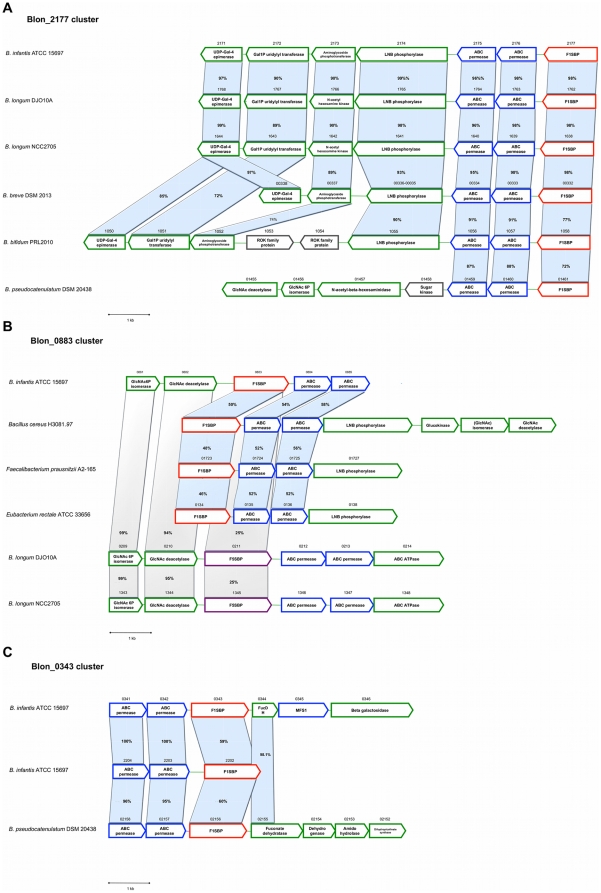
Genetic landscapes for Blon_2177 (A), Blon_0883 (B) and Blon_0343 (C), and close related homologs available in the Integrated Microbial Genome database [**72**]. Percentages represent similarity of homolog genes to those in the *B. infantis* ATCC 15697 genome. Locus tags are presented above each gene when available.

Interestingly, Blon_2177 showed an increase in its relative gene expression level with LNT and LNnT. However, this gene was not induced during growth on the HMO pool (Figures 2A and 2E) nor was it detected by shotgun proteomics (Table 2). This is expected from its HMO affinities and its gene context (Figure 3A).

Changes in gene expression during growth on GNB were not as dramatic as those induced by growth on LNT or LNnT, however Blon_2344, Blon_2350 and Blon_2351 did exhibit an increase in gene expression greater than two fold. The binding affinities for these three F1SBPs indicated that they might be involved in the import of intact mucin oligosaccharides as well of GNB.

**Figure 4:**
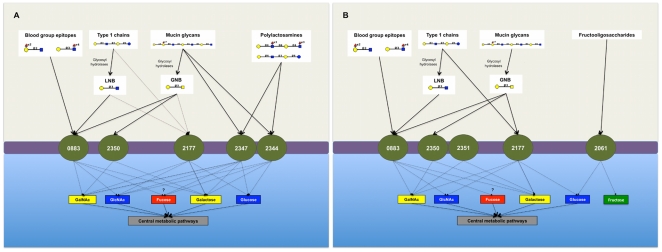
Oligosaccharide binding profile proposed for *B. infantis* cells growing on HMO (A) and inulin (B). Numbers represent each F1SBP locus tag (Blon_).

A model summarizing the complex oligosaccharide binding preferences of *B. infantis* growing on HMO is shown in Figure 4A. Cells under these conditions could import intact type 1 chains by Blon_2177, or via previous degradation by an extracellular lacto-N-biosidase to LNB to be imported by Blon_0883. Fucosylated or unfucosylated type 2 chains could also be transported in an intact form into the bacterial cell. Some mucin oligosaccharides could be imported directly into the cell, or after degradation by extracellular glycosyl hydrolases that convert these oligosaccharides into GNB. Some blood group epitopes could also be imported after release from more complex carbohydrates. The import of these molecules thereafter potentially provides the cell with monosaccharides which can be used in central metabolic pathways by this bacterium.

### F1SBP expression during growth on other prebiotics

Growth on the commercial prebiotic inulin induced the expression of seven F1SBPs (Figure 2B), however only four of these proteins were witnessed in the *B. infantis* proteome (Table 2). Several observations suggest that Blon_2061 is a candidate F1SBP for the import of fructans in *B. infantis*. Firstly, this F1SBP did not bind any glycan on the mammalian array. Additionally, the neighboring gene Blon_2056 encodes a β-fructofuranosidase, and a prominent homolog, BAD_1330, is located in a fructooligosaccharide-utilization cluster in *B. adolescentis* (Figure S1). Interestingly, Blon_2061 was the only F1SBP induced by FOS (Figure 2C). This F1SBP does not have a homolog in *B. longum* subsp. *longum* DJO10A, which suggests that oligofructose import occurs through different transport systems in both strains.

Surprisingly, inulin increased the expression of *B. infantis* F1SBPs that recognize mammalian glycans, including two proteins present in the HMO Cluster 1 with affinities for GNB, and type 1 chain binding F1SBPs Blon_2177 and Blon_0883 (Figure 2B and Table 2). These findings suggest a role for inulin inducing a cellular response directed to the intestinal environment similar to HMO, which include expression of F1SBPs involved in the import of complex intestinal glycans. Besides fructans, cells growing on inulin potentially import type 1 chains, GNB and some blood group oligosaccharides, which after digestion to mono or disaccharides by intracellular glycosyl hydrolases could be derived to glycolytic pathways (Figure 4B).

Growth on GOS induced two different F1SBPs, Blon_2414 and Blon_2458, which did not bind any of the mammalian glycans on the CFG array (Figure 2D). Blon_2414 and Blon_2458 are located close to a putative β-galactosidase (Blon_2416) and an α-galactosidase (Blon_2460), respectively (Figure S1). Blon_2458 is homolog to BL1521, a *B. longum* subsp. *longum* NCC2705 F1SBP that was induced by raffinose [37]. Finally, Blon_2444 was found to be induced by maltose (Figure S2), which is in concordance with its affinity shown by the glycan array and a similar induction of a homolog, BL0141, by maltose in *B. longum* subsp. *longum* [37].

### In vitro interactions between F1SBPs and epithelial cells

**Figure 5:**
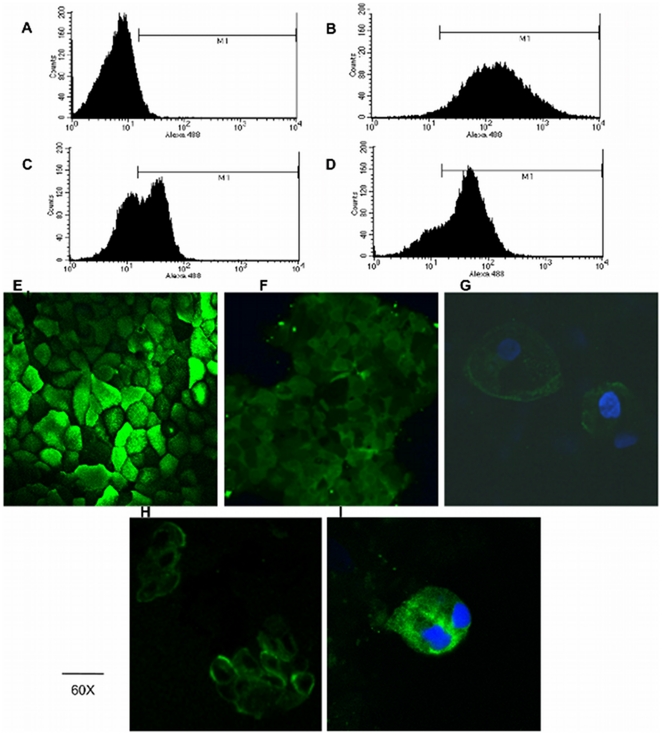
Binding of F1SBPs to Caco-2 cells *in vitro* detected by flow cytometry (A–D) and confocal microcopy (E–F). A: FITC-streptavidin; B and E: UEA-FITC 0.5 µg/ml; C: Blon_0375-FITC 1 µg/ml; D and F: Blon_2347-FITC 1 µg/ml; G: Blon_2347-FITC 1 µg/ml and DAPI (blue); H: fixed Caco-2 cells coincubated with 1 µg/ml Blon_0375-FITC; I: Caco-2 cells incubated for 10 min with 1 µg/ml Blon_0375-FITC and stained with DAPI.

Given that several of the F1SBPs exhibited adherence to glycans commonly found on the epithelial cells, we hypothesized that these binding proteins might also interact with intestinal epithelial surfaces. To test this, *B. infantis* F1SBPs with affinity for mammalian glycans (Figure 1) were labeled with FITC and coincubated with differentiated Caco-2 cells, a human colonic adenocarcinoma cell line. Using flow cytometry, a clear shift in the mean fluorescence value was observed when cells were incubated with Blon_2347 and Blon_0375 (Figures 5B–D), as well as with Ulex Europeus Agglutinin lectin (UEA). This lectin is characterized by binding oligosaccharides containing the Fucα1–2Galβ1–4GlcNAc epitope. No fluorescence was detected when cells were coincubated with other F1SBPs or FITC-streptavidin. These observations were confirmed using confocal microscopy (Figures 5E–I). Blon_2347 and Blon_0375 were found in the cell membrane of fixed Caco-2 cells. When cells were fixed after coincubation with Blon_0375, this particular protein was observed in the cytoplasm of Caco-2 cells. (Figure 5H–I). Since this protein binds globotriose, a glycolipid abundant in some cancer cell lines, it could be hypothesized that its internalization is mediated by this glycolipid in cells expressing it in their cell membranes.

## Discussion

In this work we have functionally characterized a family of proteins in *B. infantis* predicted to import oligosaccharides as part of ABC importers. Compared to related bifidobacterial genomes these genes seem to be overrepresented in this subspecies (Table 1). Using an array containing oligosaccharides abundant on mammalian cells, we determined that 10 out of 20 F1SBPs *B. infantis* have affinity for mammalian glycans, including characteristic HMO structures as well as intestinal glycoconjugates.

### Patterns of F1SBP binding to host oligosaccharides

We observed two major trends in glycan binding. Blon_0343, Blon_2202, Blon_0375, Blon_2350, Blon_2351 and Blon_2354 were specific for only one or two oligosaccharides in the glycan array (Figure 1). Considering that these substrates were not recognized by the same F1SBPs when they were present at terminal or internal positions in more complex oligosaccharides, it is likely that these proteins require their ligands to be in their free forms. It is possible that the reducing end in these carbohydrates might be critical for F1SBP binding. With the exception of Blon_0375, none of the F1SBPs listed above were able to bind epithelial Caco-2 cells, which are characterized by glycoconjugates containing these epitopes (Figure 1 and Table S2). This idea supports the role of these F1SBPs in oligosaccharide import associated to ABC transporters, and suggests a requirement on foreign extracellular glycosyl hydrolases for their release from intestinal glycoconjugates and subsequent use as a carbon source [38], [39], [40].

In contrast, Blon_0883, Blon_2177, Blon_2344 and Blon_2347 exhibited a more flexible capacity to bind either type 1 or type 2 chains on the glycan array. Because of this variable substrate binding capacity it is possible to discern a core glycan epitope interacting with the F1SBP binding pocket. An example is Blon_2177, which binds the disaccharide Galβ1–3HexNAc. Additional LNB subunits can be attached to this disaccharide structure, resulting however in a decrease in the RFU values for this protein. Blon_0883 also prefers Galβ1–3HexNAc, accepting a fucose residue in either end of the disaccharide (Figure 1).

Blon_2344 and Blon_2347 both bind dilactosamine (Galβ1–4GlcNAcβ1–3Galβ1–4GlcNAcβ) as a core structure. Modifications to this tetrasaccharide, such as additional lactosamine units, fucose residues or replacing the terminal GlcNAc with glucose did not inhibit binding. Additional studies are needed to determine to what extent the substrates bound by F1SBPs are internalized into the cell and how this interaction occurs at the structural level.

### HMO import in *B. infantis* ATCC 15697

Blon_0883 and Blon_2177, the latter present in the LNB/GNB gene cluster, were determined to bind type 1 glycans. In the glycan array Blon_0883 had affinity for LNB as well as GNB, and it was induced by HMO, LNT and LNnT. Blon_0883 is located in a gene cluster that includes two enzymes predicted to participate in hexosamine metabolism, glucosamine-6-P isomerase and glucosamine deacetylase (Figure 3B). Blon_0883 does not contain a homolog in any other bifidobacterial genome sequenced to date. Homologs are found in other members of the intestinal microbiota such as *Eubacterium rectale* and *Faecalibacterium prausnitzii*. The gene clusters in these microorganisms also contain homologs of hexosamine metabolizing enzymes, as well as a LNB phosphorylase, a key enzyme in the metabolism of type 1 chains [21]. Interestingly, *B. longum* DJO10A and NCC2705 possess a structurally similar gene cluster containing enzymes related to hexosamine metabolism (Figure 3B), however the transporter protein is a homolog to OppA proteins, predicted to import dipeptides (Family 5 of SBPs). As has been described previously for pentose utilization [41], this difference is another example of how *B. infantis* has adapted subspecies-specific mechanisms for HMO and/or mucin metabolism, but which have diverged for alternative purposes in the subspecies *longum*. In concordance with this idea, only two of the ten F1SBPs with affinity with host glycans have affinity to proteins in both *B. longum* sequenced genomes.

Blon_2177 is homologous to BL1638 in *B. longum* NCC2705. Both are located in the LNB/GNB gene cluster that is well conserved among *Bifidobacterium* species residing in the infant gastrointestinal tract (Figure 3A). Homologs to this F1SBP are present in all *B. longum* and *B. infantis* strains [42]. While BL1638 showed similar K_d_ values for GNB and LNB [43], Blon_2177 seems to prefer GNB three times more than LNB, being also able to bind other complex structures such as LNT and LNH. These structural differences may help explain the broader range of HMO structures *B. infantis* is able to consume.

Blon_2177 expression was only detected when cells grew on LNT and LNnT, and similarly to *B. longum* LMG 13197 [44], the F1SBP in this cluster was induced by inulin. Contrary to what might be predicted given its binding patterns, Blon_2177 was not expressed during growth on HMO as indicated by qRT-PCR or proteomics (Table 2). It is possible that the LNT concentration in the pooled HMO preparation is not enough to induce the expression of this protein, or certain HMO species repress its expression. Considering that both Blon_2177 and Blon_0883 bind type 1 chains, Blon_0883 might have a more active role in the import of these structures. Blon_2177 could therefore be critical in the import of non-fucosylated type 1 oligosaccharides, as well as GNB (as a product of mucin glycan degradation). In order to import type 1 HMO via Blon_0883, *B. infantis* needs to deconstruct these glycans to the disaccharide LNB. Since a lacto-N-biosidase gene has not been found in the *B. infantis* ATCC 15697 genome, it is possible that *B. infantis* relies on extracellular degradation of type 1 HMO by other intestinal bacteria.

The ability to ferment LNB seems to be present among relatively few bifidobacteria [45], and the import of this substrate has been associated so far with a single F1SBP in the LNB/GNB cluster already described. In *B. infantis* two F1SBPs were found to bind LNB and five bind GNB (Figure 1). This suggests a particular abundance of these two core structures in the infant colon, as well as a selective pressure for mechanisms to internalize them. The potential redundancy in these affinities correlates with the absence of certain F1SBPs related to HMO import in some *B. infantis* strains [42]. LNT is the main HMO isomer preferred by infant-borne bifidobacteria [24]. *B. bifidum* was recently determined to possess enzymes deconstructing type 2 chains such as LNnT [46], however the role of these glycosyl hydrolases has not been tested *in vivo* or their expression evaluated at the RNA or protein level. The *B. infantis* ATCC 15697 genome contains a unique gene cluster containing several transport related proteins and glycosyl hydrolases [27]. However as shown here, the F1SBPs in this cluster have overlapping affinities, and bind different complex type 2 chains. They can also interact with intact mucin oligosaccharides as well as the core mucin structure GNB. It is probable that this entire cluster is devoted to the deconstruction of this type of isomers, while the aforementioned clusters Blon_0883 and Blon_2177 participate in type 1 chain metabolism. These findings are in agreement with the preference for the import of complex substrates for intracellular degradation in *B. infantis* [26], contrasting with *B. breve* and *B. bifidum*, which seem to release extracellular glycosyl hydrolases to break down type 1 oligosaccharides to LNB and, potentially, metabolize type 2 oligosaccharides [26], [27]. One might predict that this strategy would be applicable for structurally similar mucin and glycolipid oligosaccharides as well.

### Impact of F1SBP expression under growth on HMO or inulin

While the binding behavior clearly predicted the types of oligosaccharides that interact with a given F1SBP and associated transport systems, the expression of these F1SBPs was not strictly governed by growth on the cognate oligosaccharide partners. Indeed, growth of *B. infantis* on inulin and HMO induced both predicted transporters involved in their own import as well as other, putatively unrelated F1SBPs. As depicted in Figure 2B, F1SBPs induced by inulin, which is a fructose-based oligosaccharide, import GNB into the cell, as well as type 1 chains and blood group glycans. HMO-induced F1SBPs were determined to bind HMO structures, but they can also import mucin oligosaccharides, and blood group structures. These observations suggest an adaptive response to two prebiotic substrates that reach the human colon, and prepare bifidobacteria to encounter other host-derived glycans. If so, an induction of other genes involved in host oligosaccharide metabolism, such as glycosyl hydrolases, could be expected. F1SBPs induced by HMO also showed binding to epithelial cells. If their binding is one of the factors involved in *B. infantis* adhesion to intestinal cells or mucins, HMO consumption could indirectly help in bacterial attachment because of F1SBPs dual function in the import and binding of intestinal oligosaccharides.

Despite their similarities, growth on inulin resulted in induction of a larger number of F1SBP than growth on FOS. This is a surprising result given these fructans differ mostly in their degree of polymerization (DP) (FOS, 2–7; inulin, 10–32). Inulin is also characterized by branched chains. Shorter chain FOS and GOS appeared to induce F1SBPs involved only in their import, in contrast to HMO and inulin which induced additional F1SBPs related to utilization, or binding, of intestinal glycans. These results suggest that different prebiotics may elicit different binding and colonization behaviors in commensal, or probiotic, bacteria *in situ*.

For some F1SBPs which affinities were determined in the glycan array, the particular genes were not induced by any of the substrates used. Blon_0343 and Blon_2202 showed affinity for 2FL and the H-disaccharide, however they were not expressed either at the transcript or protein level under the conditions studied. The localization of a fuconate dehydratase in the vicinity of Blon_0343 supports the idea of their role in the import of certain fucose containing oligosaccharides (Figure 3C). The homolog of Blon_2015 (BL1163; Fig. S1) in *B. longum* NCC2705 was induced by lactose and GOS, however this was not observed for *B. infantis* [37]. Further experiments are required to understand the role of some F1SBPs which affinities or the conditions when they are expressed are not clear. For example, Blon_2380 is homologous to BL1330, a protein that was suggested to participate in the import of mannose containing oligosaccharides [37]. Blon_0043 possesses several homologs in adult-type bifidobacteria, and it is located in the same region that Blon_0047, which is a putative xylan esterase.

### F1SBPs and their role in host-bacterial interactions

HMO share structural similarity with intestinal glycoconjugates and they prevent the binding of several pathogens to intestinal surfaces [47], [48], [49]. One competitive advantage for *B. infantis* may be the ability to both bind and consume HMO and other intestinal glycoconjugates. Thus expression of specific F1SBPs in *B. infantis* may play a role in acquisition of food substrates as well as assist in adherence to glycoconjugates on epithelial surfaces. SBPs from certain microorganisms have been found to be associated with bacterial adhesion [50], [51].

Cultured epithelial cells have been shown to be mostly unresponsive to *B. infantis* [52]. However, *B. infantis* is able to downregulate the cytokine induction profile from some gastrointestinal pathogens [53]. *B. infantis* has also been shown to secrete products that enhance epithelial cell function [54]. Candidates for adhesion and interaction with the epithelium found in other bifidobacteria or lactobacilli such as glycoprotein binding fimbriae or mucus binding proteins were not observed in the genome of *B. infantis* ATCC 15697 [27]. F1SBPs binding specific glycoconjugates in epithelial cells remain as candidates for these functions. Some oligosaccharides detected by F1SBPs are commonly found in epithelial cell membranes or occur as glycoconjugates in mucins. For example, Lewis a is one of the most abundant glycolipids in intestinal epithelial cells [55], and it was one of the ligands bound by Blon_0883. Polylactosamines are also common structures abundant in the complex type of N-linked proteins, as well as in glycolipids, especially in erythrocytes and neutrophiles [56]. Blon_2344 and Blon_2347 possessed an exquisite affinity for these structures and might be important in host-bacterial interactions. Polylactosamines are also one of the binding targets for several pathogens, such as *Helicobacter pylori* [57], and the heat-labile enterotoxin of *E. coli* [58]. Therefore, the expression of these particular F1SBPs might prevent in some cases bacterial colonization or toxin intoxication via competitive exclusion.

In the glycan array, Blon_0375 was highly specific for globotriose (Gb3), a trisaccharide usually conjugated to ceramide, and it appeared to be internalized by a discrete population of Caco-2 cells. This glycan has been found at low concentrations in human milk [59] and is characteristic of the urothelial epithelium [60] and human fetal meconium [61]. Blon_0375 binding Gb3 might provide a competitive advantage to this bacterium, and suggests that this oligosaccharide must be abundant in the environment where *B. infantis* resides. This protein could also be important in bacterial adhesion and interaction with intestinal epithelial cells. Gb3 is not abundant in adult epithelial cells, and no detailed description exists on the abundance of this glycolipid in the human intestinal mucosa in the first months of life. However, Gb3 is highly expressed in several cancer cell lines, and in particular during metastasis. Globotriose is also the target for the B-subunit of the Shiga toxin [62], and it has been successfully studied as an effector against tumors *in vivo* [63], [64].

Bifidobacteria are well-adapted microorganisms that cohabitate the lower section of the gastrointestinal tract, to which several health benefits have been attributed [65], [66]. As has been observed with some *Bacteroides* species, bifidobacteria have been characterized by their ability to utilize complex oligosaccharides, mainly plant-derived but also host-derived such as HMO [67], [68]. The existence of discrete molecular mechanisms for the import of these complex structures in bifidobacteria suggests an evolutionary adaptation to the presence of these indigestible dietary oligosaccharides in the human colon. Indeed, the induction of some common F1SBPs between inulin and HMO suggests a common regulatory mechanism. Since genomic analysis suggests *B. infantis* has displaced some plant-associated sugar catabolism genes with genes for milk sugars [41] it could be argued that the regulation of HMO consumption has simply adapted from an existing inulin-based regulon.

Defining the details of the intricate tripartite network between human milk, the developing infant intestinal epithelium and the intestinal microbiota is critical for understanding the biology of the microbial foundation process occurring in the first years of life in the infant gut, as well as for the improvement of infant formulas. In particular, understanding the nutritional preferences for complex oligosaccharides for members of the microbiota will help in the future in the selection of specific desirable probiotic strains, as well as a change towards more specific prebiotic compounds.

## Materials and Methods

### Bacteria and media

*B. longum* subsp. *infantis* ATCC 15697 was obtained from the American Type Culture Collection (Manassas, VA). Cultures were routinely grown using Mann Rogose Sharp medium with no carbon source, and supplemented with 2% w/v lactose and 0.25% w/v L-cysteine (Sigma-Aldrich, St. Louis, MO). Bacteria were routinely cultured in a vinyl anaerobic chamber (Coy Laboratory Products, Grass Lake, MI) at 37°C. Chemically competent *Escherichia coli* BL21 and DH5α were obtained from EMD Chemicals (Gibbstown, NJ), and grown in Luria Broth supplemented with 50 µg/ml ampicilin (Sigma-Aldrich, St. Louis, MO) when necessary at 37°C.

### F1SBP Cloning and Expression

Genes coding for F1SBPs were described by [27]. Gene features such as lipoprotein attachment site and signal peptide sequence were identified using the Biology workbench 3.2 (http://workbench.sdsc.edu). PCR primers were designed to target each gene excluding the signal peptide region. Primers sequences were designed with different 5′ restriction sites, and an extra six base pairs for proper enzyme restriction digestion (Table S1). Genomic DNA from *B. infantis* was purified from 2 ml cultures using the MasterPure™ Gram Positive DNA Purification Kit (Epicentre Biotechnologies, Madison, WI), following the manufacturer instructions. F1SBP genes were amplified by PCR using 0.5 µM of each forward and reverse primer, 1 ng genomic DNA, 0.2 mM dNTP mix (Fermentas, Glen Burnie, MD), and 2 U of PFU Turbo DNA polymerase (Stratagene, Cedar Creek, TX) in a 100 µl final volume. PCR was performed using a PTC200 Thermo Cycler (MJ Research, Ramsey, MN), with an initial denaturation at 95°C for 2 min, and 35 cycles of denaturation at 95°C 1 min, annealing at 58°C for 1 min, extension at 72°C 2 min, and a final extension at 72°C for 7 min. PCR products were gel purified and digested with two restriction enzymes (Fermentas, Glen Burnie, MD), which cleaved the sequences shown in bold in Table S1. The expression plasmid pGEX-6P-1 (Glutathione-S-Transferase Gene Fusion System, GE Healthcare, Piscataway, NJ), was linearized with the same two enzymes for each PCR product and dephosphorylated using Antarctic Phosphatase (New England Biolabs, Ipswich, MA), and ligated to each F1SBP gene in a 1∶3 ratio using 1 U of T4 DNA ligase (Promega, Madison, WI), in a 10 µl final volume at 4°C overnight. Ligation reactions were transformed into *E. coli* BL21 and DH5 chemically competent cells at 42°C for 30 sec and allowed to recover for 1 h at 37°C in SOC media (Invitrogen). Recombinant clones were selected for growth on LB agar with 50 µg/ml ampicilin, and confirmed by plasmid sequencing using primers pGEX 5 and pGEX 3 (GE Healthcare). Protein expression was carried out using 100 ml LB broth supplemented with 50 µg/ml ampicilin. Cells were grown at 37° in a shaker at 250 rpm (Innova-4000, New Brunswick Scientific, Edison, NJ), until they reached an O.D. of 0.6 when they were supplemented with 1 mM IPTG (Fermentas) for 2 h. In some cases due to low protein solubility, cultures were scaled to 2 L and growth was performed at 28°C. Cells were collected in an Eppendorf 5804 centrifuge (Eppendorf, Hauppauge, NY) 20 min at 4° and at 4000 rpm, and stored at −80°C at least for 18 h. Pellets were resuspended in Bugbuster Protein Extraction Reagent (EMD Chemicals), using 1 ml of the detergent for 20 ml of the original culture, and incubated for 20 min at room temperature. The suspension was centrifuged for 20 min at 13200 rpm at 4°C, and the supernatant was applied to 1 ml Bio-Scale Mini Profinity GST cartridges, connected to an EP-1 Econo-pump (Bio-Rad, Hercules, CA). Protein purification was performed as recommended by the manufacturer. Recombinant proteins were evaluated for molecular weight and purity in SDS-PAGE gels. Finally, glutathione was exchanged for PBS buffer using Amicon Ultra-15 Centrifugal Filter Units, with a cut-off of 50 kDa (Millipore, Billerica, CA).

### Glycan Array Analysis

Each GST-tagged F1SBP was screened for glycan interactions by the Core H of the Consortium for Functional Glycomics, using versions 3.1 and up of the Glycan Array. Each protein sample was diluted to 200 µg/ml in binding buffer (20 mM Tris-HCL pH 7.4, 150 mM sodium chloride, 2 mM calcium chloride, 2 mM magnesium chloride, 0.05% Tween 20 and 1% BSA), and assayed to printed glycan slides. Binding and detection were carried out using an anti-GST Alexa 488 conjugate antibody (Molecular Probes, Eugene, OR). Results were expressed as Relative Fluorescent Units (RFU), and the highest and lowest point from each set of six replicates were removed.

### RNA Extraction

*B. infantis* ATCC 15697 was grown at 37°C under anaerobic conditions on 15 ml of ZMB1 media [69], supplemented in different triplicate experiments with 2% of either lactose, glucose, fructooligosaccharides (FOS, raftilose Synergy 1, Orafti, Malvern, PA), galactooligosaccharides (GOS, Purimune, GTC Nutrition, Golden, CO), inulin (raftiline HP, Orafti, Malvern, PA), or purified HMO [70]. Also, lacto-N-tetraose, lacto-N-neotetraose and galacto-N-biose (V-Labs, Covington, LA) were supplemented at 0.5% to the ZMB media. Optical density was assayed using a PowerWave microplate spectrophotometer (BioTek Instruments, Inc., Winoosky, VT). Cultures were recovered at exponential and stationary phase, as defined by previously obtained growth curves. Cells were immediately pelleted and resuspended in 1 ml of RNA later (Ambion, Austin, TX), and stored overnight at 4°C and then at −80°C until use. Bacterial cells were washed twice with PBS buffer, and pre-lysed with 250 µl of 50 mg/ml lysozyme and 120 µl of mutanolysin at 1000 units/ml (Sigma-Aldrich). Total RNA was extracted using the Ambion RNAqueous kit. Briefly, cells were resuspended in 300 µl of Lysis/Binding solution, and vortexed for 10 sec. After adding 300 µl of 64% ethanol, the lysate was transferred to RNAse-free filter cartridges, and washed with 700 µl of wash solution 1, and twice with 500 µl of wash solution 2/3. RNA was eluted with 50 µl of elution solution twice. RNA was further purified using the Qiagen RNeasy kit. 350 µl of Buffer RLT were added to the RNA obtained, and then 250 µl ethanol were also mixed. The sample was applied to an RNeasy column centrifuged. The columns were washed with 500 µl Buffer RPE twice, and the RNA was eluted with 50 µl RNase-free water done twice. Finally, the RNA was concentrated adding 50 µl LiCl to the RNA, and incubated at −70°C for 48 h, washed with cold 70% ethanol and resuspended in 20 µl DNAse/RNAse–free water. RNA concentration was evaluated by absorbance at 260 nm (Nanodrop, Thermo Scientific, Wilmington, DE). Acceptable RNA ratios were A260/A280≥1.8, and A260/A230≥1.5. RNA was checked for integrity in an Agilent Bioanalyzer 2100 with the RNA 6000 Nano Kit (Agilent Technologies, Foster City, CA).

### Quantitative Real-Time PCR

The relative levels of gene expression for each F1SBP gene under growth with different carbon sources were evaluated by quantitative PCR. The gene Blon_0393, encoding a cysteinyl-tRNA synthetase, was chosen as an endogenous control [71]. Primers and 5′ nuclease probes were designed and synthesized by TibMolBiol (Adelphia, NJ) (Table S1). qPCR was performed in a 7500 Fast Real-Time PCR System using the Taqman One-Step RT-PCR kit (Applied Biosystems, Foster City, CA). Every reaction contained primers at 0.5 µM, each probe at 125 nM, 5 ng of RNA and Reverse Transcriptase and reaction buffer as recommended by the manufacturer. Gene expression for each gene under the different conditions representing different carbon sources was evaluated by Relative Standard Curves, both for the endogenous control and each gene on each plate. qPCR reactions were run at 48°C for 30 min, then 95°C for 10 min, and 40 cycles with denaturation at 95°C for 15 sec and annealing and elongation for 1 min at 60°C. Threshold cycle data and relative efficiencies were analyzed using the Q-Gene software **(** www.gene-quantification.de/qgene.zip **).** Gene expression levels for growth on lactose were used as the calibrator. Results were expressed as fold changes in gene expression.

### Proteomics of *B. infantis* F1SBPs

*B. infantis* cultures growing under different substrates and used for RNA extraction were also taken at exponential phase, and normalized to an OD_600_ of 1.0 by dilution or concentration. Upon centrifugation, 15 ml of each sample were washed three times with PBS vigorously. Cell pellets were resuspended in 600 µl of lysis buffer containing 100 mM Tris and 8.0 M urea. Cells were mechanically lysed using silica beads and a bead-beater (FastPrep, QBiogene, Carlsbad, CA, USA) for eight cycles of 30 s pulses each with a 30 s interval on ice. Beads and cell debris were removed by centrifugation and the soluble fraction was stored at −80°C for further analysis. Protein concentration was measured with the Protein Assay Kit (BioRad, Hercules, CA, USA). A volume of 200 mg/ml of protein was transferred to a new cap tube and precipitated by ethanol (75% (v/v)) at −20^°^C. Upon centrifugation, protein pellets were resuspended in 100 µl of 0.1 M Tris/1 M urea buffer (pH 8.0). Proteins were then digested with 5 µg of mass spectrometry grade trypsin (Promega, Madison, WI, USA) overnight at 37°C. The tryptic peptides were purified using Cap Trap Columns for peptide concentration and a Desalting Cartridge (Michrom, Aurburn, CA, USA) according to the manufacturer's manual. The peptides were eluted in 98% acetonitrile in water and then dried prior to mass spectrometry analysis.

The digested protein samples were submitted to the Genome Center Proteomics Core at the University of California, Davis. Protein identification was performed using an Eksigent Nano LC 2-D system coupled to an LTQ ion-trap mass spectrometer (Thermo-Fisher, Waltham, MI, USA) using a Picoview nano-spray source. Peptides were loaded onto a nanotrap (Zorbax 300 SB-C18, Agilent Technologies, Santa Clara, CA, USA) at a loading flow rate of 5.0 µl/min. Peptides were then eluted from the trap and separated at a level of nano-scale using a 75 µm×15 cm New Objectives Picofrit Column packed in-house. The top ten ions in each survey scan were subjected to automatic low-energy CID. Tandem mass spectra were extracted and the charge states deconvoluted by BioWorks version 3.3. All MS/MS samples were analyzed using X! Tandem (GPM-XE manager, ver. 2.2.1). X! Tandem was set up to search against the *B. infantis* ATCC 15697 whole proteome. X! Tandem was searched with a fragment ion mass tolerance of 0.60 Da. Oxidation of methionine was specified as a variable modification in X! Tandem. Cutoff of log(e) for the peptide (log(e)) was set < −2 and the protein with the log(e) <−6 was considered to be present.

### F1SBP *in vitro* binding to Caco-2 cells

Recombinant F1SBPs were purified as described previously, and labeled using the Fluoreporter FITC Protein Labeling Kit (Molecular Probes) as recommended by the manufacturer. Caco-2 cells were obtained from the American Type Culture Collection, and routinely cultured in DMEM supplemented with 10% Fetal Bovine Serum, 10 mM HEPES and 1% Penicillin-Streptomycin (Invitrogen, Carlsbad, CA) on 75 cm^2^ flasks and incubated at 37° with 5% CO_2_. Cells were routinely passed to new flasks using 0.25% trypsin (Invitrogen) two weeks after confluence. For flow cytometry experiments, trypsinized Caco-2 cells were fixed in 4% paraformaldehyde (PFA, Sigma-Aldrich), and after two washes with PBS, they were incubated with different concentrations of labeled F1SBPs in PBS buffer or UEA-FITC lectin (Vector laboratories, Burlingame, CA) at 37°C for 1 h. Cells were centrifuged at 3000 x *g* 4 min, and washed four times with PBS-tween 0.05%. Lectin-labeled cells were analyzed by flow cytometry using a Becton Dickinson FACScan Flow Cytometer. 30000 events for each sample were recorded using FSC, SSC and an argon laser at 488 nm emission. Fluorescence was measured at 515/540 nm excitation, and analyzed using the CellQuest software. For confocal microscopy, Caco-2 cells were seeded in eight-well chamber slides (Nalgene Nunc International, Naperville, IL), and after two weeks post-confluence they were washed with PBS and fixed with PFA 4% for 20 min. After two washes with PBS, monolayers were incubated with different concentrations of FITC-labeled F1SBPs or UEA-FITC lectin, and incubated at 37°C 1 h. After four washes with PBS 1X, slides were permeabilized with 0.1% Triton X-100 and incubated with 0.1 ng/ml 4′,6-diamidino-2-phenylindole (DAPI, Invitrogen, Carlsbad, CA), and finally covered with VectaShield Mounting Medium (Vector laboratories, Burlingame, CA). In some cases Caco-2 monolayers were incubated with F1SBPs for 10 or 30 minutes and then cells were fixed and processed as indicated. Slides were analyzed with an Olympus FV1000 Laser Scanning Confocal microscope (Olympus, Melville, NY).

## Supporting Information

Figure S1 **Genetic landscapes for other F1SBP clusters.** A: Blon_2015; B: Blon_2061; C: Blon_2414; D: Blon_2468.
(PDF)

Figure S2 **Fold change in gene expression of Blon_2444 in** ***B. infantis*** **cells grown in maltose (2%) relative to lactose.**
(PDF)

Table S1 **Primers used in this study.**
(DOC)

Table S2 **List of oligosaccharides in the Mammalian Glycan Array v3.1.**
(DOCX)
