# Supplementary figures and images for: Oligosaccharide Binding Proteins from Bifidobacterium longum subsp. infantis Reveal a Preference for Host Glycans
Source: PLoS One. 2011 Mar 15;6(3):e17315. doi: 10.1371/journal.pone.0017315 (PMC3057974; doi:10.1371/journal.pone.0017315)

A

Blon\_2015 cluster

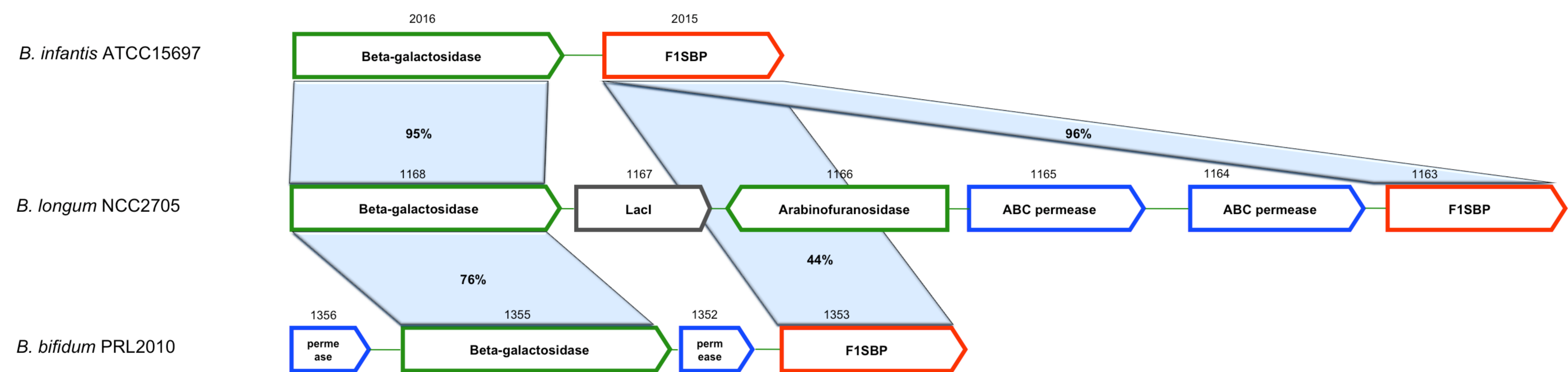

B

Blon\_2061 cluster

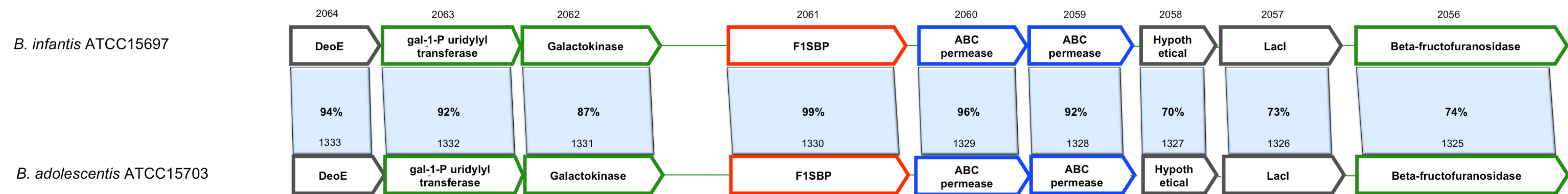

C

Blon\_2414 cluster

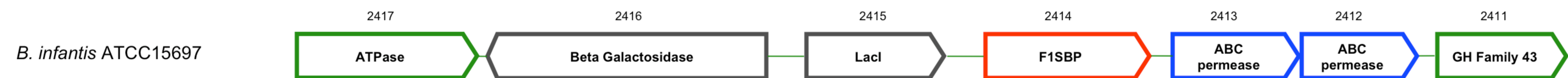

D

Blon\_2458 cluster

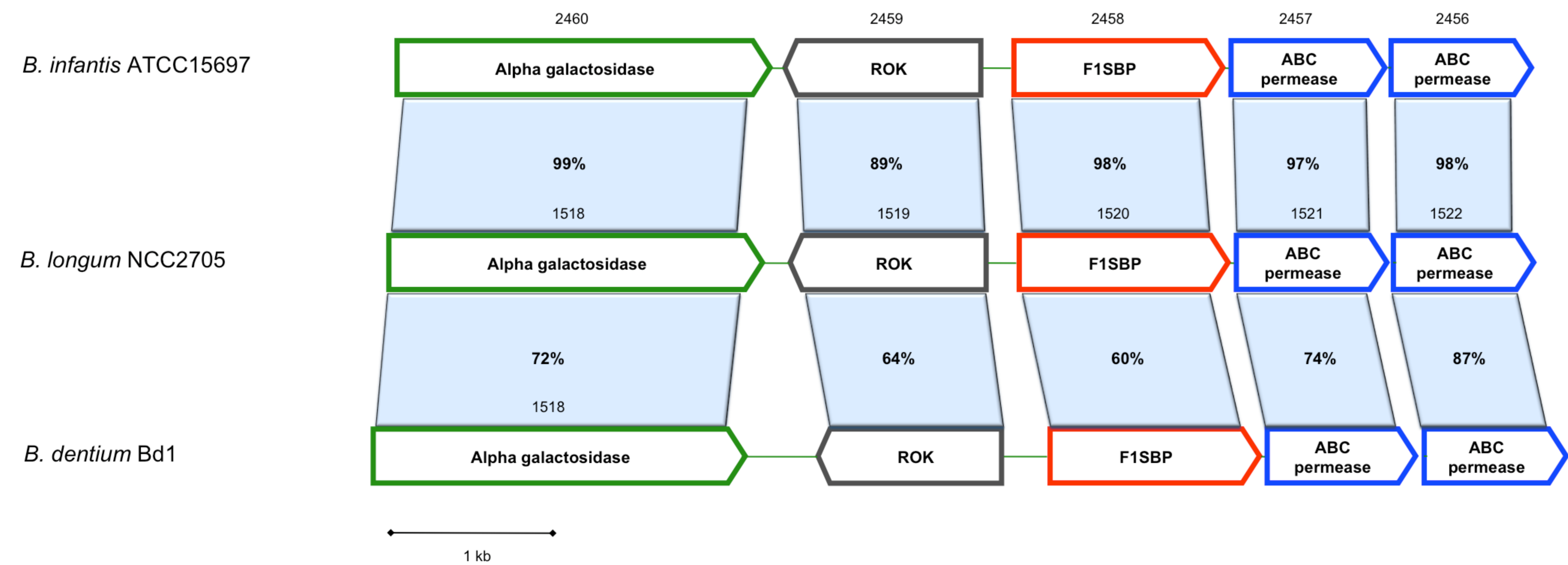

Supplement: Figure S1 — Genetic landscapes for other F1SBP clusters. A: Blon_2015; B: Blon_2061; C: Blon_2414; D: Blon_2468. (PDF) [file pone.0017315.s001.pdf]

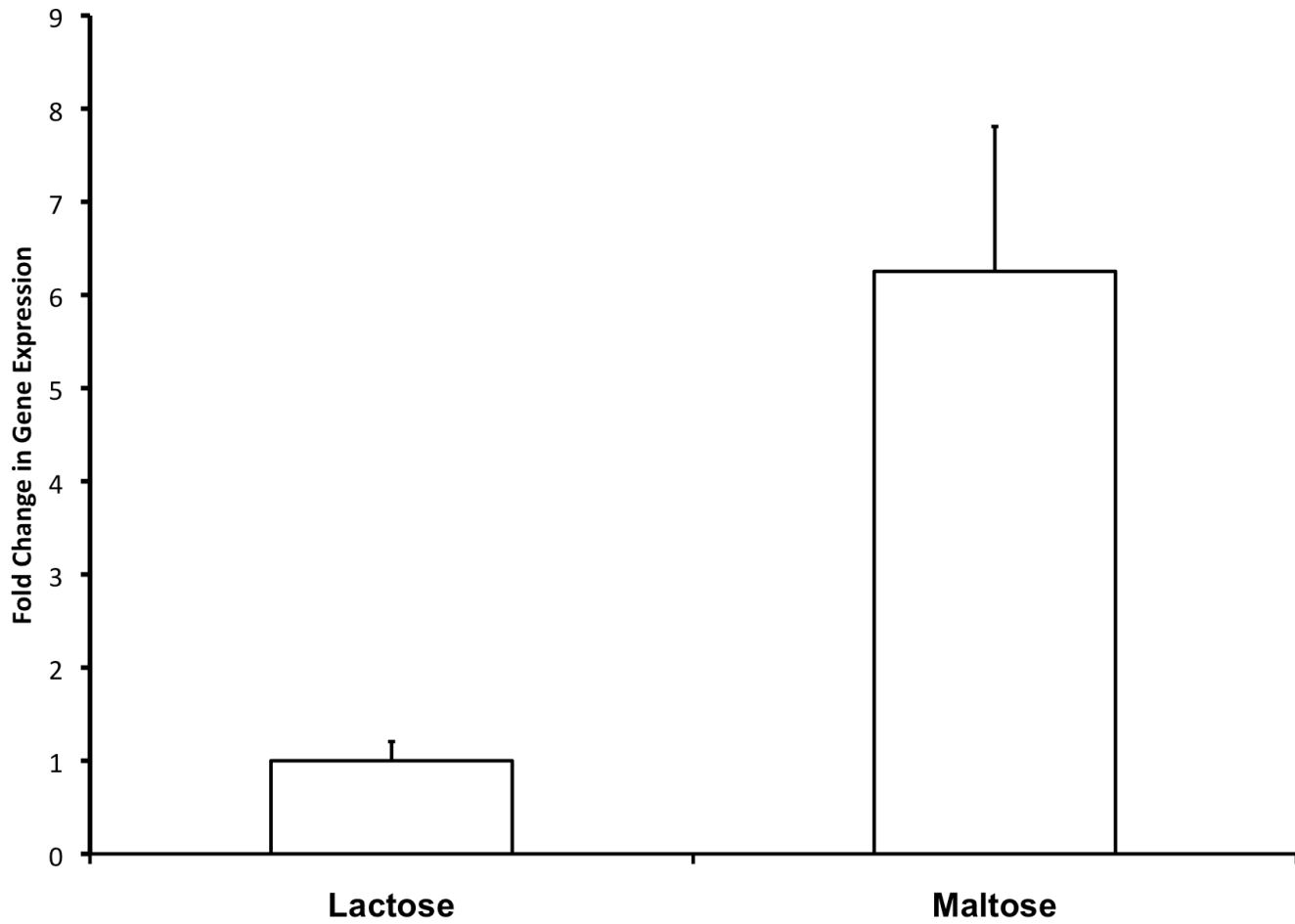

Supplement: Figure S2 — Fold change in gene expression of Blon_2444 in B. infantis cells grown in maltose (2%) relative to lactose. (PDF) [file pone.0017315.s002.pdf]
