# Supplementary material for: Oligosaccharide Binding Proteins from Bifidobacterium longum subsp. infantis Reveal a Preference for Host Glycans
Source: PLoS One. 2011 Mar 15;6(3):e17315. doi: 10.1371/journal.pone.0017315 (PMC3057974; doi:10.1371/journal.pone.0017315)
Supplement: Table S1 — Primers used in this study. (DOC) [file pone.0017315.s003.doc]

**Table S1:** Primers used in this study.

*F1SBP cloning*

**Primer Sequence (5’-3’)**

BL0043F^a^ ATAT**CCCGGG**CAGCGACACCGCGCAAGATGA

BL0043R ATAT**CTCGAG**TCAGTTGGTCTTGACGCCCATGTCTT

BL0343F ATAT**GTCGAC**GCTCTGGTACTTCGCAGAAAAACAA

BL0343R ATAT**GCGGCCGC**TTTCAGTCTGCGTCAGTGGTGACTTTA

BL0375F ATAT**GTCGAC**GCGGGAAAACCAAGATTTCGTTCT

BL0375R ATAT**GCGGCCGC**AGTTACTTCAGCGTCGCC

BL0883F ATAT**GAATTC**TCCGATGGCGGCAAGACCACACTCAAATTC

BL0883R ATAT**CTCGAG**TTACTGCTTGGCCGCCGCGTTCA

BL2015w TGCGAGTGATCGGACAGCGAATC

BL2015F ATAT**CTCGAG**GGACGACAACCGTACGGAGATC

BL2015R ATAT**CGGCCG**CTACTACTTAATCGTAAAGCCC

BL2061F ATAT**CCCGGG**CGAGAACGGCAAGCCAATCGTCAA

BL2061R ATAT**GCGGCCGC**CTCTACTTGGTGTACTTGTCGTAC

BL2177F ATAT**GAATTC**ACGAAGTCCGGCAGCGATGGCGG

BL2177R ATAT**GCGGCCGC**CTTCACTCCTTGACGGACAGAC

BL2202F ATAT**GTCGAC**GCGATGTGACCGCGCAGGACGT

BL2202R ATAT**CTCGAG**TCAGTCGGCGTCGGTGGTGACCTTG

BL2344w ATGAGAAGAACCGCGATGAGG

BL2344F ATATGTCGACGCGAAGGAACCCAATCAGGACAAGA

BL2344R ATAT**GCGGCCGC**TCACTTCTTCGTGTAGATGTCGTA

BL2347F ATAT**GAATTC**GACGGCAAGCCGATCGTGAGCGTTC

BL2347R ATAT**GCGGCCGC**CTTCACTTCTTCGTGTAGATGTCG

BL2347w CGCACTGAAGGCGGGCGCCATCAC

BL2350F ATAT**GTCGAC**ATGGCAAGCCGATTGTAAGTGTCTT

BL2350R ATAT**CTCGAG**TCACTTCGTGTAGGTGTCGTACCAC

BL2350w GCTGTCTGCGTGCGGCGGCGA

BL2351F ATAT**GAATTC**GGCAAGCCGATTGTGACGGTTCTGGTC

BL2351R ATAT**GCGGCCGC**CTTCACTTCGTGTAGGTGTCGT

BL2351w GTGAAGGCGGGTGCCGTTGCGTGC

BL2352F ATAT**GTCGAC**ACGGCAAGCCGATTGTGACGGTTCTGGTCA

BL2352R ATAT**GCGGCCGC**CTTCACTTTACGCAAAGGTCGTACCA

BL2352w GTGAAGGCGGGTGCCGTTGTGTGT

BL2354F ATAT**GAATTC**GGCAAGCCGATTGTGACGGTTCTGGTC

BL2354R ATAT**GCGGCCGC**CTTCACTTTACGCAAAGGTCGTA

BL2354w CGATGAAGGTGGGTGCTGCGGTA

BL2357F ATAT**GAATTC**GATGGTGGACAATCCGACAAGATCGTCTCC

BL2357R ATAT**CTCGAG**TCACTGTTCCCAATCCGAATCGAACGTC

BL2367F ATAT**GAATTC**GTCCCTACGCCCAAAGAGTCCGACGGTT

BL2367R ATAT**CTCGAG**TCACTTCTGGGACAGCGAGTTATTGTAA

BL2380F ATAT**GTCGAC**ACGGTAAGAAAGAAGTCTCCTTCCAGACCT

BL2380R ATAT**CTCGAG**TCATGAGTTCAAGTCCTCATTGGCGATC

BL2414F ATAT**CCCGGG**CGCCGGCAAGATCCGGCTCA

BL2414R ATAT**GAATTC**TCACTGCGCGGCCGCGACCTTG

BL2444F ATAT**GTCGAC**GAAGCTCGACCTCCGGCGATGACGC

BL2444R ATAT**GCGGCCGC**CTTCAGTGCGAGGCCTCGTATT

BL2458F ATAT**GTCGAC**CCGTTACGCTGGATTTCTTCCAGTTCAAG

BL2458R ATAT**GCGGCCGC**CTTCACTCGAAGGTCCTGGCTTGGACC

*qPCR*

**Primer Sequence (5’-3’)**

Blon_0043F TACGCCTCCTACGCCAAT

Blon_0043R CGTACTTCTCGTGGATACCCT

Blon_0043TM 6FAM-TTCTTGGTGCCGTTCACCAGTTCCT-BBQ

Blon_0343F TTTGAAGGGTAAGATGGTCGTC

Blon_0343R TGCTGCCATTGACCCAC

Blon_0343TM 6FAM-AAGCCAATGCCGCTGCTTGCTTAT-BBQ

Blon_0375F GAAGACGGCCAGTGGATC

Blon_0375R AGGAGGTTCGGCATTGTACT

Blon_0375TM 6FAM-AGGCTTCGCCATCGCGTCCA-BBQ

Blon_0393F TTCACCGAGGCGTACAACA

Blon_0393R CGCATCCGTGACCACATAG

Blon_0393TM 6FAM-AGAATGCGCTGAATCAGGTCGATCAT-BBQ

Blon_0883F ATCGAAGCCGTGTGGATT

Blon_0883R CCTCGTTGTAGGCGTCGTA

Blon_0883TM 6FAM-ACACCTTCATGTCGGAGGCCAGGT-BBQ

Blon_2015F CCCGTCATTTCCGTGTGAT

Blon_2015R CCACGTATTCTTTAGGGTCGTAG

Blon_2015TM 6FAM-CATCCAGCGTTGCCTCCATGC-BBQ

Blon_2061F GGTCCAACTACGCCGAATA

Blon_2061R CGTACATGGCGTCGATCA

Blon_2061TM 6FAM-CTTGGCGACCAGTACGTCACCCT-PH

Blon_2177 F GGTTCCTGAGGTCTTCACCA

Blon_2177 R GCCGAGCTTCTCAAATTCA

Blon_2177 TM 6FAM-AGTACAAGGACGATTTCGCTTCCGC-PH

Blon_2202F ATGAGAACCAGCGCAATAAG

Blon_2202R CAGATCGCCGTTCTCATTC

Blon_2202TM 6FAM-TCCGGCACGATGACATCGTTGAAC-BBQ

Blon_2344F TCAAGAAGCTCGACCCGTTG

Blon_2344R TTGGCGTAGAAGCCGTATGT

Blon_2344TM 6FAM- ACTACACCTGGCACAGCCCGATGCT-BBQ

Blon_2347F AAGCCGATAGGTTCTCCCT

Blon_2347R TCGCCTTGGTGTACTTGTCT

Blon_2347TM 6FAM-AGCTGGCCAACCTGCTCTACTCCGA-BBQ

Blon_2350F GGTCTGTCTGATCGGTTTACG

Blon_2350R CTGCGCTGCTCATCATATG

Blon_2350TM 6FAM-TCCCGACGAAGTCTCCATTAAGGGC-BBQ

Blon_2351F GTTCGGCAGCTTGTCAAGA

Blon_2351R AGTGGCAGGAAGTGACTCG

Blon_2351TM 6FAM-AACGCCACCTTGGCCTCTGGAGATA-BBQ

Blon_2352F GCGCCCTGTAGCTATCGAA

Blon_2352R GACGTGAACCTGAATGGATACG

Blon_2352TM 6FAM-ACAAGCTGCCAAACGTCAAGGCATT-BBQ

Blon_2354F ACTGATAGGCGTCAAGGGAA

Blon_2354R GGACAAGATCGCCAATATGC

Blon_2354TM 6FAM-AGGACTTGGAGGCCGACTGCGAT-BBQ

Blon_2357F GCGGACTTACGAACAGGAAT

Blon_2357R GCGGCCTATCTGTATGAGAAG

Blon_2357TM 6FAM-ACGTCCAAGGACAGCCAGATTGAGT-BBQ

Blon_2367F GTAGTTCCCCACACCGACT

Blon_2367R CAACATCGGCATCACCAT

Blon_2367TM 6FAM- AATCCACCGCCGATCTGGCC-BBQ

Blon_2380 F CAGGATCAGGCCGTCAA

Blon_2380 R TCATTGGCGATCTTGACGA

Blon_2380 TM 6FAM-AAGGCCGTGCTCGACGATGACTG--BBQ

Blon_2414F GTTCGCTCTTGAGAATGTCCG

Blon_2414R ACCAGAAGGTCTACGATCAGG

Blon_2414TM 6FAM-CGGCAAGTACTACCTGCACACCAAC-BBQ

Blon_2444F GACTTCCTCAAGTGGGTGATC

Blon_2444R ACGGCATCATGGTGAAGTT
Blon_2444TM 6FAM-AACCTGGGTCTTGCCGGACTCCT—BBQ

Blon_2458F TCTACGACTTCACCGATGAGC

Blon_2458R AGCATGTCGGTGAACTCG

Blon_2458TM 6FAM-TCGAGACCTACCTTGCGGAACAGC--BBQ

^a^ Sequence in bold represent the restriction site for each restriction enzyme
