# Supplementary material for: Oligosaccharide Binding Proteins from Bifidobacterium longum subsp. infantis Reveal a Preference for Host Glycans
Source: PLoS One. 2011 Mar 15;6(3):e17315. doi: 10.1371/journal.pone.0017315 (PMC3057974; doi:10.1371/journal.pone.0017315)
Supplement: Table S2 — List of oligosaccharides in the Mammalian Glycan Array v3.1. (DOCX) [file pone.0017315.s004.docx]

| **Chart #** | **Masterlist Name** |
| --- | --- |
| 1 | Neu5Acα2-8Neu5Acβ-Sp17 |
| 2 | Neu5Acα2-8Neu5Acα2-8Neu5Acβ-Sp8 |
| 3 | Neu5Gcβ2-6Galβ1-4GlcNAc-Sp8 |
| 4 | Galβ1-3GlcNAcβ1-2Manα1-3(Galβ1-3GlcNAcβ1-2Manα1-6)Manβ1-4GlcNAcβ1-4GlcNAcβ-Sp19 |
| 5 | Galα-Sp8 |
| 6 | Glcα-Sp8 |
| 7 | Manα-Sp8 |
| 8 | GalNAcα-Sp8 |
| 9 | Fucα-Sp8 |
| 10 | Fucα-Sp9 |
| 11 | Rha-Sp8 |
| 12 | Neu5Acα-Sp8 |
| 13 | Neu5Acα-Sp11 |
| 14 | Neu5Acβ-Sp8 |
| 15 | Galβ-Sp8 |
| 16 | Glcβ-Sp8 |
| 17 | Manβ-Sp8 |
| 18 | GalNAcβ-Sp8 |
| 19 | GlcNAcβ-Sp0 |
| 20 | GlcNAcβ-Sp8 |
| 21 | GlcN(Gc)β-Sp8 |
| 22 | Galβ1-4GlcNAcβ1-3(Galβ1-4GlcNAcβ1-6)GalNAcα-Sp8 |
| 23 | GlcNAcβ1-3(GlcNAcβ1-4)(GlcNAcβ1-6)GlcNAc-Sp8 |
| 24 | [3OSO3][6OSO3]Galβ1-4[6OSO3]GlcNAcβ-Sp0 |
| 25 | [3OSO3][6OSO3]Galβ1-4GlcNAcβ-Sp0 |
| 26 | [3OSO3]Galβ1-4Glcβ-Sp8 |
| 27 | [3OSO3]Galβ1-4[6OSO3]Glcβ-Sp0 |
| 28 | [3OSO3]Galβ1-4[6OSO3]Glcβ-Sp8 |
| 29 | [3OSO3]Galβ1-3(Fucα1-4)GlcNAcβ-Sp8 |
| 30 | [3OSO3]Galβ1-3GalNAcα-Sp8 |
| 31 | [3OSO3]Galβ1-3GlcNAcβ-Sp8 |
| 32 | [3OSO3]Galβ1-4(Fucα1-3)GlcNAcβ-Sp8 |
| 33 | [3OSO3]Galβ1-4[6OSO3]GlcNAcβ-Sp8 |
| 34 | [3OSO3]Galβ1-4GlcNAcβ-Sp0 |
| 35 | [3OSO3]Galβ1-4GlcNAcβ-Sp8 |
| 36 | [3OSO3]Galβ-Sp8 |
| 37 | [4OSO3][6OSO3]Galβ1-4GlcNAcβ-Sp0 |
| 38 | [4OSO3]Galβ1-4GlcNAcβ-Sp8 |
| 39 | 6-H2PO3Manα-Sp8 |
| 40 | [6OSO3]Galβ1-4Glcβ-Sp0 |
| 41 | [6OSO3]Galβ1-4Glcβ-Sp8 |
| 42 | [6OSO3]Galβ1-4GlcNAcβ-Sp8 |
| 43 | [6OSO3]Galβ1-4[6OSO3]Glcβ-Sp8 |
| 44 | Neu5Acα2-3[6OSO3]Galβ1-4GlcNAcβ-Sp8 |
| 45 | [6OSO3]GlcNAcβ-Sp8 |
| 46 | Neu5Ac(9Ac)α-Sp8 |
| 47 | Neu5Ac(9Ac)α2-6Galβ1-4GlcNAcβ-Sp8 |
| 48 | Manα1-3(Manα1-6)Manβ1-4GlcNAcβ1-4GlcNAcβ-Sp13 |
| 49 | GlcNAcβ1-2Manα1-3(GlcNAcβ1-2Manα1-6)Manβ1-4GlcNAcβ1-4GlcNAcβ-Sp13 |
| 50 | Galβ1-4GlcNAcβ1-2Manα1-3(Galβ1-4GlcNAcβ1-2Manα1-6)Manβ1-4GlcNAcβ1-4GlcNAcβ-Sp12 |
| 51 | Galβ1-4GlcNAcβ1-2Manα1-3(Galβ1-4GlcNAcβ1-2Manα1-6)Manβ1-4GlcNAcβ1-4GlcNAcβ-Sp13 |
| 52 | Neu5Acα2-6Galβ1-4GlcNAcβ1-2Manα1-3(Neu5Acα2-6Galβ1-4GlcNAcβ1-2Manα1-6)Manβ1-4GlcNAcβ1-4GlcNAcβ-Sp12 |
| 53 | Neu5Acα2-6Galβ1-4GlcNAcβ1-2Manα1-3(Neu5Acα2-6Galβ1-4GlcNAcβ1-2Manα1-6)Manβ1-4GlcNAcβ1-4GlcNAcβ-Sp13 |
| 54 | Neu5Acα2-6Galβ1-4GlcNAcβ1-2Manα1-3(Neu5Acα2-6Galβ1-4GlcNAcβ1-2Manα1-6)Manβ1-4GlcNAcβ1-4GlcNAcβ-Sp8 |
| 55 | Fucα1-2Galβ1-3GalNAcβ1-3Galα-Sp9 |
| 56 | Fucα1-2Galβ1-3GalNAcβ1-3Galα1-4Galβ1-4Glcβ-Sp9 |
| 57 | Fucα1-2Galβ1-3(Fucα1-4)GlcNAcβ-Sp8 |
| 58 | Fucα1-2Galβ1-3GalNAcα-Sp8 |
| 59 | Fucα1-2Galβ1-3GalNAcβ1-4(Neu5Acα2-3)Galβ1-4Glcβ-Sp0 |
| 60 | Fucα1-2Galβ1-3GalNAcβ1-4(Neu5Acα2-3)Galβ1-4Glcβ-Sp9 |
| 61 | Fucα1-2Galβ1-3GlcNAcβ1-3Galβ1-4Glcβ-Sp10 |
| 62 | Fucα1-2Galβ1-3GlcNAcβ1-3Galβ1-4Glcβ-Sp8 |
| 63 | Fucα1-2Galβ1-3GlcNAcβ-Sp0 |
| 64 | Fucα1-2Galβ1-3GlcNAcβ-Sp8 |
| 65 | Fucα1-2Galβ1-4(Fucα1-3)GlcNAcβ1-3Galβ1-4(Fucα1-3)GlcNAcβ-Sp0 |
| 66 | Fucα1-2Galβ1-4(Fucα1-3)GlcNAcβ1-3Galβ1-4(Fucα1-3)GlcNAcβ1-3Galβ1-4(Fucα1-3)GlcNAcβ-Sp0 |
| 67 | Fucα1-2Galβ1-4(Fucα1-3)GlcNAcβ-Sp0 |
| 68 | Fucα1-2Galβ1-4(Fucα1-3)GlcNAcβ-Sp8 |
| 69 | Fucα1-2Galβ1-4GlcNAcβ1-3Galβ1-4GlcNAcβ-Sp0 |
| 70 | Fucα1-2Galβ1-4GlcNAcβ1-3Galβ1-4GlcNAcβ1-3Galβ1-4GlcNAcβ-Sp0 |
| 71 | Fucα1-2Galβ1-4GlcNAcβ-Sp0 |
| 72 | Fucα1-2Galβ1-4GlcNAcβ-Sp8 |
| 73 | Fucα1-2Galβ1-4Glcβ-Sp0 |
| 74 | Fucα1-2Galβ-Sp8 |
| 75 | Fucα1-3GlcNAcβ-Sp8 |
| 76 | Fucα1-4GlcNAcβ-Sp8 |
| 77 | Fucb1-3GlcNAcβ-Sp8 |
| 78 | GalNAcα1-3(Fucα1-2)Galβ1-3GlcNAcβ-Sp0 |
| 79 | GalNAcα1-3(Fucα1-2)Galβ1-4(Fucα1-3)GlcNAcβ-Sp0 |
| 80 | GalNAcα1-3(Fucα1-2)Galβ1-4GlcNAcβ-Sp0 |
| 81 | GalNAcα1-3(Fucα1-2)Galβ1-4GlcNAcβ-Sp8 |
| 82 | GalNAcα1-3(Fucα1-2)Galβ1-4Glcβ-Sp0 |
| 83 | GalNAcα1-3(Fucα1-2)Galβ-Sp8 |
| 84 | GalNAcα1-3GalNAcβ-Sp8 |
| 85 | GalNAcα1-3Galβ-Sp8 |
| 86 | GalNAcα1-4(Fucα1-2)Galβ1-4GlcNAcβ-Sp8 |
| 87 | GalNAcβ1-3GalNAcα-Sp8 |
| 88 | GalNAcβ1-3(Fucα1-2)Galβ-Sp8 |
| 89 | GalNAcβ1-3Galα1-4Galβ1-4GlcNAcβ-Sp0 |
| 90 | GalNAcβ1-4(Fucα1-3)GlcNAcβ-Sp0 |
| 91 | GalNAcβ1-4GlcNAcβ-Sp0 |
| 92 | GalNAcβ1-4GlcNAcβ-Sp8 |
| 93 | Galα1-2Galβ-Sp8 |
| 94 | Galα1-3(Fucα1-2)Galβ1-3GlcNAcβ-Sp0 |
| 95 | Galα1-3(Fucα1-2)Galβ1-4(Fucα1-3)GlcNAcβ-Sp0 |
| 96 | Galα1-3(Fucα1-2)Galβ1-4GlcNAc-Sp0 |
| 97 | Galα1-3(Fucα1-2)Galβ1-4Glcβ-Sp0 |
| 98 | Galα1-3(Fucα1-2)Galβ-Sp8 |
| 99 | Galα1-3(Galα1-4)Galβ1-4GlcNAcβ-Sp8 |
| 100 | Galα1-3GalNAcα-Sp8 |
| 101 | Galα1-3GalNAcβ-Sp8 |
| 102 | Galα1-3Galβ1-4(Fucα1-3)GlcNAcβ-Sp8 |
| 103 | Galα1-3Galβ1-3GlcNAcβ-Sp0 |
| 104 | Galα1-3Galβ1-4GlcNAcβ-Sp8 |
| 105 | Galα1-3Galβ1-4Glcβ-Sp0 |
| 106 | Galα1-3Galβ-Sp8 |
| 107 | Galα1-4(Fucα1-2)Galβ1-4GlcNAcβ-Sp8 |
| 108 | Galα1-4Galβ1-4GlcNAcβ-Sp0 |
| 109 | Galα1-4Galβ1-4GlcNAcβ-Sp8 |
| 110 | Galα1-4Galβ1-4Glcβ-Sp0 |
| 111 | Galα1-4GlcNAcβ-Sp8 |
| 112 | Galα1-6Glcβ-Sp8 |
| 113 | Galβ1-2Galβ-Sp8 |
| 114 | Galβ1-3(Fucα1-4)GlcNAcβ1-3Galβ1-4(Fucα1-3)GlcNAcβ-Sp0 |
| 115 | Galβ1-3(Fucα1-4)GlcNAcβ1-3Galβ1-4GlcNAcβ-Sp0 |
| 116 | Galβ1-3(Fucα1-4)GlcNAcβ-Sp0 |
| 117 | Galβ1-3(Fucα1-4)GlcNAc-Sp8 |
| 118 | Galβ1-3(Fucα1-4)GlcNAcβ-Sp8 |
| 119 | Galβ1-3(Galβ1-4GlcNAcβ1-6)GalNAcα-Sp8 |
| 120 | Galβ1-3(GlcNAcβ1-6)GalNAcα-Sp8 |
| 121 | Galβ1-3(Neu5Acα2-6)GalNAcα-Sp8 |
| 122 | Galβ1-3(Neu5Acβ2-6)GalNAcα-Sp8 |
| 123 | Galβ1-3(Neu5Acα2-6)GlcNAcβ1-4Galβ1-4Glcβ-Sp10 |
| 124 | Galβ1-3GalNAcα-Sp8 |
| 125 | Galβ1-3GalNAcβ-Sp8 |
| 126 | Galβ1-3GalNAcβ1-3Galα1-4Galβ1-4Glcβ-Sp0 |
| 127 | Galβ1-3GalNAcβ1-4(Neu5Acα2-3)Galβ1-4Glcβ-Sp0 |
| 128 | Galβ1-3GalNAcβ1-4Galβ1-4Glcβ-Sp8 |
| 129 | Galβ1-3Galβ-Sp8 |
| 130 | Galβ1-3GlcNAcβ1-3Galβ1-4GlcNAcβ-Sp0 |
| 131 | Galβ1-3GlcNAcβ1-3Galβ1-4Glcβ-Sp10 |
| 132 | Galβ1-3GlcNAcβ-Sp0 |
| 133 | Galβ1-3GlcNAcβ-Sp8 |
| 134 | Galβ1-4(Fucα1-3)GlcNAcβ-Sp0 |
| 135 | Galβ1-4(Fucα1-3)GlcNAcβ-Sp8 |
| 136 | Galβ1-4(Fucα1-3)GlcNAcβ1-4Galβ1-4(Fucα1-3)GlcNAcβ-Sp0 |
| 137 | Galβ1-4(Fucα1-3)GlcNAcβ1-4Galβ1-4(Fucα1-3)GlcNAcβ1-4Galβ1-4(Fucα1-3)GlcNAcβ-Sp0 |
| 138 | Galβ1-4[6OSO3]Glcβ-Sp0 |
| 139 | Galβ1-4[6OSO3]Glcβ-Sp8 |
| 140 | Galβ1-4GalNAcα1-3(Fucα1-2)Galβ1-4GlcNAcβ-Sp8 |
| 141 | Galβ1-4GalNAcβ1-3(Fucα1-2)Galβ1-4GlcNAcβ-Sp8 |
| 142 | Neu5Acα2-3Galβ1-4GlcNAcβ1-2Manα1-3(Neu5Acα2-3Galβ1-4GlcNAcβ1-2Manα1-6)Manβ1-4GlcNAcβ1-4GlcNAcβ-Sp12 |
| 143 | Galβ1-4GlcNAcβ1-3GalNAcα-Sp8 |
| 144 | Galβ1-4GlcNAcβ1-3Galβ1-4(Fucα1-3)GlcNAcβ1-3Galβ1-4(Fucα1-3)GlcNAcβ-Sp0 |
| 145 | Galβ1-4GlcNAcβ1-3Galβ1-4GlcNAcβ1-3Galβ1-4GlcNAcβ-Sp0 |
| 146 | Galβ1-4GlcNAcβ1-3Galβ1-4GlcNAcβ-Sp0 |
| 147 | Galβ1-4GlcNAcβ1-3Galβ1-4Glcβ-Sp0 |
| 148 | Galβ1-4GlcNAcβ1-3Galβ1-4Glcβ-Sp8 |
| 149 | Galβ1-4GlcNAcβ1-6(Galβ1-3)GalNAcα-Sp8 |
| 150 | Galβ1-4GlcNAcβ1-6GalNAcα-Sp8 |
| 151 | Galβ1-4GlcNAcβ-Sp0 |
| 152 | Galβ1-4GlcNAcβ-Sp8 |
| 153 | Galβ1-4Glcβ-Sp0 |
| 154 | Galβ1-4Glcβ-Sp8 |
| 155 | GlcNAcα1-3Galβ1-4GlcNAcβ-Sp8 |
| 156 | GlcNAcα1-6Galβ1-4GlcNAcβ-Sp8 |
| 157 | GlcNAcβ1-2Galβ1-3GalNAcα-Sp8 |
| 158 | GlcNAcβ1-3(GlcNAcβ1-6)GalNAcα-Sp8 |
| 159 | GlcNAcβ1-3(GlcNAcβ1-6)Galβ1-4GlcNAcβ-Sp8 |
| 160 | GlcNAcβ1-3GalNAcα-Sp8 |
| 161 | GlcNAcβ1-3Galβ-Sp8 |
| 162 | GlcNAcβ1-3Galβ1-3GalNAcα-Sp8 |
| 163 | GlcNAcβ1-3Galβ1-4GlcNAcβ-Sp0 |
| 164 | GlcNAcβ1-3Galβ1-4GlcNAcβ-Sp8 |
| 165 | GlcNAcβ1-3Galβ1-4GlcNAcβ1-3Galβ1-4GlcNAcβ-Sp0 |
| 166 | GlcNAcβ1-3Galβ1-4Glcβ-Sp0 |
| 167 | GlcNAcβ1-4-MDPLys |
| 168 | GlcNAcβ1-4(GlcNAcβ1-6)GalNAcα-Sp8 |
| 169 | GlcNAcβ1-4Galβ1-4GlcNAcβ-Sp8 |
| 170 | (GlcNAcβ1-4)6b-Sp8 |
| 171 | (GlcNAcβ1-4)5b-Sp8 |
| 172 | GlcNAcβ1-4GlcNAcβ1-4GlcNAcβ-Sp8 |
| 173 | GlcNAcβ1-6(Galβ1-3)GalNAcα-Sp8 |
| 174 | GlcNAcβ1-6GalNAcα-Sp8 |
| 175 | GlcNAcβ1-6Galβ1-4GlcNAcβ-Sp8 |
| 176 | Glcα1-4Glcβ-Sp8 |
| 177 | Glcα1-4Glcα-Sp8 |
| 178 | Glcα1-6Glcα1-6Glcβ-Sp8 |
| 179 | Glcβ1-4Glcβ-Sp8 |
| 180 | Glcβ1-6Glcβ-Sp8 |
| 181 | G-ol-Sp8 |
| 182 | Glcαa-Sp8 |
| 183 | Glcαb-Sp8 |
| 184 | Glcαb1-3Galβ-Sp8 |
| 185 | Glcαb1-6Galβ-Sp8 |
| 186 | KDNa2-3Galβ1-3GlcNAcβ-Sp0 |
| 187 | KDNa2-3Galβ1-4GlcNAcβ-Sp0 |
| 188 | Manα1-2Manα1-2Manα1-3Manα-Sp9 |
| 189 | Manα1-2Manα1-3(Manα1-2Manα1-6)Manα-Sp9 |
| 190 | Manα1-2Manα1-3Manα-Sp9 |
| 191 | Manα1-6(Manα1-2Manα1-3)Manα1-6(Manα1-2Manα1-3)Manβ1-4GlcNAcβ1-4GlcNAcβ-Sp12 |
| 192 | Manα1-2Manα1-6(Manα1-3)Manα1-6(Manα1-2Manα1-2Manα1-3)Manβ1-4GlcNAcβ1-4GlcNAcβ-Sp12 |
| 193 | Manα1-2Manα1-2Manα1-3(Manα1-2Manα1-3(Manα1-2Manα1-6)Manα1-6)Manβ1-4GlcNAcβ1-4GlcNAcβ-Sp12 |
| 194 | Manα1-3(Manα1-6)Manα-Sp9 |
| 195 | Manα1-3(Manα1-2Manα1-2Manα1-6)Manα-Sp9 |
| 196 | Manα1-6(Manα1-3)Manα1-6(Manα1-2Manα1-3)Manβ1-4GlcNAcβ1-4GlcNAcβ-Sp12 |
| 197 | Manα1-6(Manα1-3)Manα1-6(Manα1-3)Manβ1-4GlcNAcβ1-4 GlcNAcβ-Sp12 |
| 198 | Neu5Acα2-6Galβ1-4GlcNAcβ1-2Manα1-3(Neu5Acα2-3Galβ1-4GlcNAcβ1-2Manα1-6)Manβ1-4GlcNAcβ1-4GlcNAcβ-Sp12 |
| 199 | Manβ1-4GlcNAcβ-Sp0 |
| 200 | Fucα1-3(Galβ1-4)GlcNAcβ1-2Manα1-3(Fucα1-3(Galβ1-4)GlcNAcβ1-2Manα1-6)Manβ1-4GlcNAcβ1-4GlcNAcβ-Sp20 |
| 201 | Neu5Acα2-3Galβ1-3GalNAcα-Sp8 |
| 202 | Neu5Acα2-8Neu5Acα2-8Neu5Acα2-8Neu5Acα2-3(GalNAcβ1-4)Galβ1-4Glcβ-Sp0 |
| 203 | Neu5Acα2-8Neu5Acα2-8Neu5Acα2-3(GalNAcβ1-4)Galβ1-4Glcβ-Sp0 |
| 204 | Neu5Acα2-8Neu5Acα2-8Neu5Acα2-3Galβ1-4Glcβ-Sp0 |
| 205 | Neu5Acα2-8Neu5Acα2-3(GalNAcβ1-4)Galβ1-4Glcβ-Sp0 |
| 206 | Neu5Acα2-8Neu5Acα2-8Neu5Acα-Sp8 |
| 207 | Neu5Acα2-3(6-O-Su)Galβ1-4(Fucα1-3)GlcNAcβ-Sp8 |
| 208 | Neu5Acα2-3(GalNAcβ1-4)Galβ1-4GlcNAcβ-Sp0 |
| 209 | Neu5Acα2-3(GalNAcβ1-4)Galβ1-4GlcNAcβ-Sp8 |
| 210 | Neu5Acα2-3(GalNAcβ1-4)Galβ1-4Glcβ-Sp0 |
| 211 | Neu5Acα2-3(Neu5Acα2-3Galβ1-3GalNAcβ1-4)Galβ1-4Glcβ-Sp0 |
| 212 | Neu5Acα2-3(Neu5Acα2-6)GalNAcα-Sp8 |
| 213 | Neu5Acα2-3GalNAcα-Sp8 |
| 214 | Neu5Acα2-3GalNAcβ1-4GlcNAcβ-Sp0 |
| 215 | Neu5Acα2-3Galβ1-3[6OSO3]GlcNAc-Sp8 |
| 216 | Neu5Acα2-3Galβ1-3(Fucα1-4)GlcNAcβ-Sp8 |
| 217 | Neu5Acα2-3Galβ1-3(Fucα1-4)GlcNAcβ1-3Galβ1-4(Fucα1-3)GlcNAcβ-Sp0 |
| 218 | Neu5Acα2-3Galβ1-3(Neu5Acα2-3Galβ1-4)GlcNAcβ-Sp8 |
| 219 | Neu5Acα2-3Galβ1-3[6OSO3]GalNAcα-Sp8 |
| 220 | Neu5Acα2-3Galβ1-3(Neu5Acα2-6)GalNAcα-Sp8 |
| 221 | Neu5Acα2-3Galβ-Sp8 |
| 222 | Neu5Acα2-3Galβ1-3GalNAcβ1-3Galα1-4Galβ1-4Glcβ-Sp0 |
| 223 | Neu5Acα2-3Galβ1-3GlcNAcβ1-3Galβ1-4GlcNAcβ-Sp0 |
| 224 | Neu5Acα2-3Galβ1-3GlcNAcβ-Sp0 |
| 225 | Neu5Acα2-3Galβ1-3GlcNAcβ-Sp8 |
| 226 | Neu5Acα2-3Galβ1-4[6OSO3]GlcNAcβ-Sp8 |
| 227 | Neu5Acα2-3Galβ1-4(Fucα1-3)[6OSO3]GlcNAcβ-Sp8 |
| 228 | Neu5Acα2-3Galβ1-4(Fucα1-3)GlcNAcβ1-3Galβ1-4(Fucα1-3)GlcNAcβ1-3Galβ1-4(Fucα1-3)GlcNAcβ-Sp0 |
| 229 | Neu5Acα2-3Galβ1-4(Fucα1-3)GlcNAcβ-Sp0 |
| 230 | Neu5Acα2-3Galβ1-4(Fucα1-3)GlcNAcβ-Sp8 |
| 231 | Neu5Acα2-3Galβ1-4(Fucα1-3)GlcNAcβ1-3Galβ-Sp8 |
| 232 | Neu5Acα2-3Galβ1-4(Fucα1-3)GlcNAcβ1-3Galβ1-4GlcNAcβ-Sp8 |
| 233 | Neu5Acα2-3Galβ1-4GlcNAcβ1-3Galβ1-4(Fucα1-3)GlcNAc-Sp0 |
| 234 | Neu5Acα2-3Galβ1-4GlcNAcβ1-3Galβ1-4GlcNAcβ1-3Galβ1-4GlcNAcβ-Sp0 |
| 235 | Neu5Acα2-3Galβ1-4GlcNAcβ-Sp0 |
| 236 | Neu5Acα2-3Galβ1-4GlcNAcβ-Sp8 |
| 237 | Neu5Acα2-3Galβ1-4GlcNAcβ1-3Galβ1-4GlcNAcβ-Sp0 |
| 238 | Neu5Acα2-3Galβ1-4Glcβ-Sp0 |
| 239 | Neu5Acα2-3Galβ1-4Glcβ-Sp8 |
| 240 | Neu5Acα2-6GalNAcα-Sp8 |
| 241 | Neu5Acα2-6GalNAcβ1-4GlcNAcβ-Sp0 |
| 242 | Neu5Acα2-6Galβ1-4[6OSO3]GlcNAcβ-Sp8 |
| 243 | Neu5Acα2-6Galβ1-4GlcNAcβ-Sp0 |
| 244 | Neu5Acα2-6Galβ1-4GlcNAcβ-Sp8 |
| 245 | Neu5Acα2-6Galβ1-4GlcNAcβ1-3Galβ1-4(Fucα1-3)GlcNAcβ1-3Galβ1-4(Fucα1-3)GlcNAcβ-Sp0 |
| 246 | Neu5Acα2-6Galβ1-4GlcNAcβ1-3Galβ1-4GlcNAcβ-Sp0 |
| 247 | Neu5Acα2-6Galβ1-4Glcβ-Sp0 |
| 248 | Neu5Acα2-6Galβ1-4Glcβ-Sp8 |
| 249 | Neu5Acα2-6Galβ-Sp8 |
| 250 | Neu5Acα2-8Neu5Acα-Sp8 |
| 251 | Neu5Acα2-8Neu5Acα2-3Galβ1-4Glcβ-Sp0 |
| 252 | Neu5Acβ2-6GalNAcα-Sp8 |
| 253 | Neu5Acβ2-6Galβ1-4GlcNAcβ-Sp8 |
| 254 | Neu5Gca2-3Galβ1-3(Fucα1-4)GlcNAcβ-Sp0 |
| 255 | Neu5Gca2-3Galβ1-3GlcNAcβ-Sp0 |
| 256 | Neu5Gca2-3Galβ1-4(Fucα1-3)GlcNAcβ-Sp0 |
| 257 | Neu5Gca2-3Galβ1-4GlcNAcβ-Sp0 |
| 258 | Neu5Gca2-3Galβ1-4Glcβ-Sp0 |
| 259 | Neu5Gca2-6GalNAcα-Sp0 |
| 260 | Neu5Gca2-6Galβ1-4GlcNAcβ-Sp0 |
| 261 | Neu5Gca-Sp8 |
| 262 | [3OSO3]Galβ1-4(Fucα1-3)[6OSO3]Glc-Sp0 |
| 263 | [3OSO3]Galβ1-4(Fucα1-3)Glc-Sp0 |
| 264 | [3OSO3]Galβ1-4(Fucα1-3)[6OSO3]GlcNAc-Sp8 |
| 265 | [3OSO3]Galβ1-4(Fucα1-3)GlcNAc-Sp0 |
| 266 | Fucα1-2[6OSO3]Galβ1-4GlcNAc-Sp0 |
| 267 | Fucα1-2Galβ1-4[6OSO3]GlcNAc-Sp8 |
| 268 | Fucα1-2[6OSO3]Galβ1-4[6OSO3]Glc-Sp0 |
| 269 | Fucα1-2[6OSO3]Galβ1-4Glc-Sp0 |
| 270 | Fucα1-2Galβ1-4[6OSO3]Glc-Sp0 |
| 271 | Galβ1-3(Fucα1-4)GlcNAcβ1-3Galβ1-3(Fucα1-4)GlcNAcβ-Sp0 |
| 272 | Galβ1-3(Galβ1-4GlcNAcβ1-6)GalNAc-Sp14 |
| 273 | Galβ1-3(GlcNAcβ1-6)GalNAc-Sp14 |
| 274 | Galβ1-3(Neu5Acα2-3Galβ1-4GlcNAcβ1-6)GalNAcα-Sp14 |
| 275 | Galβ1-3GalNAcα-Sp14 |
| 276 | Galβ1-3GlcNAcβ1-3Galβ1-3GlcNAcβ-Sp0 |
| 277 | Galβ1-4(Fucα1-3)[6OSO3]GlcNAc-Sp0 |
| 278 | Galβ1-4(Fucα1-3)[6OSO3]Glc-Sp0 |
| 279 | Galβ1-4(Fucα1-3)GlcNAcβ1-3Galβ1-3(Fucα1-4)GlcNAcβ-Sp0 |
| 280 | Galβ1-4GlcNAcβ1-3Galβ1-3GlcNAcβ-Sp0 |
| 281 | Neu5Acα2-3Galβ1-3GlcNAcβ1-3Galβ1-3GlcNAcβ-Sp0 |
| 282 | Neu5Acα2-3Galβ1-4GlcNAcβ1-3Galβ1-3GlcNAcβ-Sp0 |
| 283 | [3OSO3]Galβ1-4[6OSO3]GlcNAcβ-Sp0 |
| 284 | [3OSO3][4OSO3]Galβ1-4GlcNAcβ-Sp0 |
| 285 | [6OSO3]Galβ1-4[6OSO3]GlcNAcβ-Sp0 |
| 286 | 6-H2PO3Glcβ-Sp10 |
| 287 | Galα1-3(Fucα1-2)Galβ-Sp18 |
| 288 | Galα1-3GalNAcα-Sp16 |
| 289 | Galβ1-3GalNAcα-Sp16 |
| 290 | Galβ1-3(Neu5Acα2-3Galβ1-4(Fucα1-3)GlcNAcβ1-6)GalNAcα-Sp14 |
| 291 | Galβ1-3Galβ1-4GlcNAcβ-Sp8 |
| 292 | Galβ1-4GlcNAcβ1-2Manα1-3(Neu5Acα2-6Galβ1-4GlcNAcβ1-2Manα1-6)Manβ1-4GlcNAcβ1-4GlcNAcβ-Sp12 |
| 293 | Galβ1-4GlcNAcβ1-3(Galβ1-4GlcNAcβ1-6)Galβ1-4GlcNAc-Sp0 |
| 294 | Galβ1-4GlcNAcβ1-3(GlcNAcβ1-6)Galβ1-4GlcNAc-Sp0 |
| 295 | Galβ1-4GlcNAcα1-6Galβ1-4GlcNAcβ-Sp0 |
| 296 | Galβ1-4GlcNAcβ1-6Galβ1-4GlcNAcβ-Sp0 |
| 297 | GalNAcα1-3(Fucα1-2)Galβ-Sp18 |
| 298 | GalNAcα-Sp15 |
| 299 | GalNAcβ1-3Galβ-Sp8 |
| 300 | Glcαb1-3GlcNAcβ-Sp8 |
| 301 | GlcNAcβ1-2Manα1-3(Neu5Acα2-6Galβ1-4GlcNAcβ1-2Manα1-6)Manβ1-4GlcNAcβ1-4GlcNAcβ-Sp12 |
| 302 | GlcNAcβ1-2Manα1-3(GlcNAcβ1-2Manα1-6)Manβ1-4GlcNAcβ1-4GlcNAcβ-Sp12 |
| 303 | GlcNAcβ1-3Man-Sp10 |
| 304 | GlcNAcβ1-4GlcNAcβ-Sp10 |
| 305 | GlcNAcβ1-4GlcNAcβ-Sp12 |
| 306 | HOOC(CH3)CH-3-O-GlcNAcβ1-4GlcNAcβ-Sp10 |
| 307 | Manα1-3(Manα1-6)Manβ1-4GlcNAcβ1-4GlcNAcβ-Sp12 |
| 308 | Manα1-6Manβ-Sp10 |
| 309 | Manα1-6(Manα1-3)Manα1-6(Manα1-3)Manβ-Sp10 |
| 310 | Manα1-2Manα1-2Manα1-3(Manα1-2Manα1-6(Manα1-3)Manα1-6)Manα-Sp9 |
| 311 | Manα1-2Manα1-2Manα1-3(Manα1-2Manα1-6(Manα1-2Manα1-3)Manα1-6)Manα-Sp9 |
| 312 | Neu5Acα2-3Galβ1-3(Neu5Acα2-3Galβ1-4GlcNAcβ1-6)GalNAcα-Sp14 |
| 313 | Neu5Acα2-3Galβ1-3(Neu5Acα2-6)GalNAcα-Sp14 |
| 314 | Neu5Acα2-3Galβ1-3GalNAcα-Sp14 |
| 315 | Neu5Acα2-3Galβ1-4GlcNAcβ1-2Manα1-3(Neu5Acα2-6Galβ1-4GlcNAcβ1-2Manα1-6)Manβ1-4GlcNAcβ1-4GlcNAcβ-Sp12 |
| 316 | Neu5Acα2-6Galβ1-4GlcNAcβ1-2Manα1-3(Galβ1-4GlcNAcβ1-2Manα1-6)Manβ1-4GlcNAcβ1-4GlcNAcβ-Sp12 |
| 317 | Neu5Acα2-6Galβ1-4GlcNAcβ1-2Manα1-3(GlcNAcβ1-2Manα1-6)Manβ1-4GlcNAcβ1-4GlcNAcβ-Sp12 |
| 318 | Neu5Acα2-6Galβ1-4GlcNAcβ1-2Manα1-3(Neu5Acα2-6Galβ1-4GlcNAcβ1-2Manα1-6)Manβ1-4GlcNAcβ1-4GlcNAcβ-N(LT)AVL |
| 319 | Fucα1-2Galβ1-3GalNAcα-Sp14 |
| 320 | Galβ1-3(Neu5Acα2-6)GalNAcα-Sp14 |
| 321 | Galβ1-4GlcNAcβ1-3GalNAc-Sp14 |
| 322 | Neu5Ac(9Ac)a2-3Galβ1-4GlcNAcβ-Sp0 |
| 323 | Neu5Ac(9Ac)a2-3Galβ1-3GlcNAcβ-Sp0 |
| 324 | Neu5Acα2-6Galβ1-4GlcNAcβ1-3Galβ1-3GlcNAcβ-Sp0 |
| 325 | Neu5Acα2-3Galβ1-3(Fucα1-4)GlcNAcβ1-3Galβ1-3(Fucα1-4)GlcNAcβ-Sp0 |
| 326 | Neu5Acα2-6Galβ1-4GlcNAcβ1-3Galβ1-4GlcNAcβ1-3Galβ1-4GlcNAcβ-Sp0 |
| 327 | Galα1-4Galβ1-4GlcNAcβ1-3Galβ1-4Glcβ-Sp0 |
| 328 | GalNAcβ1-3Galα1-4Galβ1-4GlcNAcβ1-3Galβ1-4Glcβ-Sp0 |
| 329 | GalNAcα1-3(Fucα1-2)Galβ1-4GlcNAcβ1-3Galβ1-4GlcNAcβ-Sp0 |
| 330 | GalNAcα1-3(Fucα1-2)Galβ1-4GlcNAcβ1-3Galβ1-4GlcNAcβ1-3Galβ1-4GlcNAcβ-Sp0 |
| 331 | (Neu5Acα2-3-Galβ1-3)(((Neu5Acα2-3-Galβ1-4(Fucα1-3))GlcNAcβ1-6)GalNAc-Sp14 |
| 332 | GlcNAcα1-4Galβ1-4GlcNAcβ1-3Galβ1-4GlcNAcβ1-3Galβ1-4GlcNAcβ-Sp0 |
| 333 | GlcNAcα1-4Galβ1-4GlcNAcβ-Sp0 |
| 334 | GlcNAcα1-4Galβ1-3GlcNAcβ-Sp0 |
| 335 | GlcNAcα1-4Galβ1-4GlcNAcβ1-3Galβ1-4Glcβ-Sp0 |
| 336 | GlcNAcα1-4Galβ1-4GlcNAcβ1-3Galβ1-4(Fucα1-3)GlcNAcβ1-3Galβ1-4(Fucα1-3)GlcNAcβ-Sp0 |
| 337 | GlcNAcα1-4Galβ1-4GlcNAcβ1-3Galβ1-4GlcNAcβ-Sp0 |
| 338 | GlcNAcα1-4Galβ1-3GalNAc-Sp14 |
| 339 | Manα1-3(Neu5Acα2-6Galβ1-4GlcNAcβ1-2Manα1-6)Manβ1-4GlcNAcβ1-4GlcNAc-Sp12 |
| 340 | Neu5Acα2-6Galβ1-4GlcNAcβ1-2Manα1-3(Manα1-6)Manβ1-4GlcNAcβ1-4GlcNAc-Sp12 |
| 341 | Neu5Acα2-6Galβ1-4GlcNAcβ1-2Manα1-6Manβ1-4GlcNAcβ1-4GlcNAc-Sp12 |
| 342 | Neu5Acα2-6Galβ1-4GlcNAcβ1-2Manα1-3Manβ1-4GlcNAcβ1-4GlcNAc-Sp12 |
| 343 | Galβ1-4GlcNAcβ1-2Manα1-3Manβ1-4GlcNAcβ1-4GlcNAc-Sp12 |
| 344 | Galβ1-4GlcNAcβ1-2Manα1-6Manβ1-4GlcNAcβ1-4GlcNAc-Sp12 |
| 345 | Galβ1-4GlcNAcβ1-2Manα1-3(Manα1-6)Manβ1-4GlcNAcβ1-4GlcNAcβ-Sp12 |
| 346 | GlcNAcβ1-2Manα1-3(GlcNAcβ1-2Manα1-6)Manβ1-4GlcNAcβ1-4(Fucα1-6)GlcNAcβ-Sp22 |
| 347 | Galβ1-4GlcNAcβ1-2Manα1-3(Galβ1-4GlcNAcβ1-2Manα1-6)Manβ1-4GlcNAcβ1-4(Fucα1-6)GlcNAcβ-Sp22 |
| 348 | Galβ1-3GlcNAcβ1-2Manα1-3(Galβ1-3GlcNAcβ1-2Manα1-6)Manβ1-4GlcNAcβ1-4(Fucα1-6)GlcNAcβ-Sp22 |
| 349 | Galβ1-3(Fucα1-4)GlcNAcβ1-2Manα1-3(Galβ1-3(Fucα1-4)GlcNAcβ1-2Manα1-6)Manβ1-4GlcNAcβ1-4GlcNAcβ-Sp19 |
| 350 | [6OSO3]GlcNAcβ1-3Gal b1-4GlcNAc-b-Sp0 |
| 351 | KDNa2-3Galβ1-4(Fucα1-3)GlcNAc-Sp0 |
| 352 | KDNa2-6Galβ1-4GlcNAc-Sp0 |
| 353 | KDNa2-3Galβ1-4Glc-Sp0 |
| 354 | KDNa2-3Galβ1-3GalNAcα-Sp14 |
| 355 | Fucα1-2Galβ1-3GlcNAcβ1-2Manα1-3(Fucα1-2Galβ1-3GlcNAcβ1-2Manα1-6)Manβ1-4GlcNAcβ1-4GlcNAcβ-Sp20 |
| 356 | Fucα1-2Galβ1-4GlcNAcβ1-2Manα1-3(Fucα1-2Galβ1-4GlcNAcβ1-2Manα1-6)Manβ1-4GlcNAcβ1-4GlcNAcβ-Sp20 |
| 357 | Fucα1-2Galβ1-4(Fucα1-3)GlcNAcβ1-2Manα1-3(Fucα1-2Galβ1-4(Fucα1-3)GlcNAcβ1-2Manα1-6)Manβ1-4GlcNAcβ1-4GlcNAb-Sp20 |
| 358 | Galα1-3Galβ1-4GlcNAcβ1-2Manα1-3(Galα1-3Galβ1-4GlcNAcβ1-2Manα1-6)Manβ1-4GlcNAcβ1-4GlcNAcβ-Sp20 |
| 359 | Manα1-3(Galβ1-4GlcNAcβ1-2Manα1-6)Manβ1-4GlcNAcβ1-4GlcNAcβ-Sp12 |
| 360 | Galβ1-3(Fucα1-4)GlcNAcβ1-2Manα1-3(Galβ1-3(Fucα1-4)GlcNAcβ1-2Manα1-6)Manβ1-4GlcNAcβ1-4(Fucα1-6)GlcNAcβ-Sp22 |
| 361 | Neu5Acα2-6GlcNAcβ1-4GlcNAc-Sp21 |
| 362 | Neu5Acα2-6GlcNAcβ1-4GlcNAcβ1-4GlcNAc-Sp21 |
| 363 | Fucα1-2Galβ1-3GlcNAcβ1-3(Galβ1-4(Fucα1-3)GlcNAcβ1-6)Galβ1-4Glc-Sp21 |
| 364 | Galβ1-4GlcNAcβ1-2(Galβ1-4GlcNAcβ1-4)Manα1-3(Galβ1-4GlcNAcβ1-2Manα1-6)Manβ1-4GlcNAcβ1-4GlcNAc-Sp21 |
| 365 | GalNAcα1-3(Fucα1-2)Galβ1-4GlcNAcβ1-2Manα1-3(GalNAcα1-3(Fucα1-2)Galβ1-4GlcNAcβ1-2Manα1-6)Manβ1-4GlcNAcβ1-4GlcNAcβ-Sp20 |
| 366 | Galα1-3(Fucα1-2)Galβ1-4GlcNAcβ1-2Manα1-3(Galα1-3(Fucα1-2)Galβ1-4GlcNAcβ1-2Manα1-6)Manβ1-4GlcNAcβ1-4GlcNAcβ-Sp20 |
| 367 | Galα1-3Galβ1-4(Fucα1-3)GlcNAcβ1-2Manα1-3(Galα1-3Galβ1-4(Fucα1-3)GlcNAcβ1-2Manα1-6)Manβ1-4GlcNAcβ1-4GlcNAcβ-Sp20 |
| 368 | GalNAcα1-3(Fucα1-2)Galβ1-3GlcNAcβ1-2Manα1-3(GalNAcα1-3(Fucα1-2)Galβ1-3GlcNAcβ1-2Manα1-6)Manβ1-4GlcNAcβ1-4GlcNAcβ-Sp20 |
| 369 | Galα1-3(Fucα1-2)Galβ1-3GlcNAcβ1-2Manα1-3(Galα1-3(Fucα1-2)Galβ1-3GlcNAcβ1-2Manα1-6)Manβ1-4GlcNAcβ1-4GlcNAcβ-Sp20 |
| 370 | Fucα1-2Galβ1-3(Fucα1-4)GlcNAcβ1-2Manα1-3(Fucα1-2Galβ1-3(Fucα1-4)GlcNAcβ1-2Manα1-6)Manβ1-4GlcNAcβ1-4GlcNAcβ-Sp19 |
| 371 | NeuAca2-3Galβ1-4GlcNAcβ1-3GalNAc-Sp14 |
| 372 | NeuAca2-6Galβ1-4GlcNAcβ1-3GalNAc-Sp14 |
| 373 | Neu5Acα2-3Galβ1-4(Fucα1-3)GlcNAcβ1-3GalNAcα-Sp14 |
| 374 | GalNAcβ1-4GlcNAcβ1-2Manα1-6(GalNAcβ1-4GlcNAcβ1-2Manα1-6)Manβ1-4GlcNAcβ1-4GlcNAc-Sp12 |
| 375 | Galβ1-3GalNAcα1-3(Fucα1-2)Galβ1-4Glc-Sp14 |
| 376 | Galβ1-3GalNAcα1-3(Fucα1-2)Galβ1-4GlcNAc-Sp14 |
| 377 | GlcNAcβ1-3GalNAcα-Sp14 |
| 378 | GlcNAcβ1-6GalNAcα-Sp14 |
| 379 | Galβ1-3GlcNAcβ1-3(Galβ1-3GlcNAcβ1-3Galβ1-4GlcNAcβ1-6)Galβ1-4Glcβ-Sp0 |
| 380 | Galβ1-3GlcNAcβ1-3(Galβ1-4(Fucα1-3)GlcNAcβ1-6)Galβ1-4Glc-Sp21 |
| 381 | Fucα1-2Galβ1-3(Fucα1-4)GlcNAcβ1-3(Galβ1-4GlcNAcβ1-6)Galβ1-4Glc-Sp21 |
| 382 | Fucα1-2Galβ1-3(Fucα1-4)GlcNAcβ1-3(Galβ1-4(Fucα1-3)GlcNAcβ1-6)Galβ1-4Glc-Sp21 |
| 383 | Galβ1-3GlcNAcβ1-3(Galβ1-3GlcNAcβ1-3Galβ1-4(Fucα1-3)GlcNAcβ1-6)Galβ1-4Glc-Sp21 |
| 384 | Galβ1-4GlcNAcβ1-2(Galβ1-4GlcNAcβ1-4)Manα1-3(Galβ1-4GlcNAcβ1-2(Galβ1-4GlcNAcβ1-6)Manα1-6)Manβ1-4GlcNAcβ1-4GlcNAcβ-Sp21 |
| 385 | GlcNAcβ1-2(GlcNAcβ1-4)Manα1-3(GlcNAcβ1-2Manα1-6)Manβ1-4GlcNAcβ1-4GlcNac-Sp21 |
| 386 | Fucα1-2Galβ1-3GalNAcα1-3(Fucα1-2)Galβ1-4Glcβ-Sp0 |
| 387 | Fucα1-2Galβ1-3GalNAcα1-3(Fucα1-2)Galβ1-4GlcNAcβ-Sp0 |
| 388 | Galβ1-3GlcNAcβ1-3GalNAcα-Sp14 |
| 389 | Neu5Acα2-3(GalNAcβ1-4)Galβ1-4GlcNAcβ1-3GalNAcα-Sp14 |
| 390 | GalNAcα1-3(Fucα1-2)Galβ1-3GalNAcα1-3(Fucα1-2)Galβ1-4GlcNAcβ-Sp0 |
| 391 | Galα1-3Galβ1-3GlcNAcβ1-2Manα1-3(Galα1-3Galβ1-3GlcNAcβ1-2Manα1-6)Manβ1-4GlcNAcβ1-4GlcNAc-Sp19 |
| 392 | Galα1-3Galβ1-3(Fucα1-4)GlcNAcβ1-2Manα1-3(Galα1-3Galβ1-3(Fucα1-4)GlcNAcβ1-2Manα1-6)Manβ1-4GlcNAcβ1-4GlcNAc-Sp19 |
| 393 | Neu5Acα2-3Galβ1-3GlcNAcβ1-2Manα1-3(Neu5Acα2-3Galβ1-3GlcNAcβ1-2Manα1-6)Manβ1-4GlcNAcβ1-4GlcNAc-Sp19 |
| 394 | Galβ1-4GlcNAcβ1-2Manα1-3(GlcNAcβ1-2Manα1-6)Manβ1-4GlcNAcβ1-4GlcNAc-Sp12 |
| 395 | GlcNAcβ1-2Manα1-3(Galβ1-4GlcNAcβ1-2Manα1-6)Manβ1-4GlcNAcβ1-4GlcNAc-Sp12 |
| 396 | Neu5Acα2-3Galβ1-3GlcNAcβ1-3GalNAcα-Sp14 |
| 397 | Fucα1-2Galβ1-4GlcNAcβ1-3GalNAcα-Sp14 |
| 398 | Galβ1-4(Fucα1-3)GlcNAcβ1-3GalNAcα-Sp14 |
| 399 | GalNAcα1-3GalNAcβ1-3Galα1-4Galβ1-4GlcNAcβ-Sp0 |
| 400 | Galα1-4Galβ1-3GlcNAcβ1-2Manα1-3(Galα1-4Galβ1-3GlcNAcβ1-2Manα1-6)Manβ1-4GlcNAcβ1-4GlcNAcβ-Sp19 |
| 401 | Galα1-4Galβ1-4GlcNAcβ1-2Manα1-3(Galα1-4Galβ1-4GlcNAcβ1-2Manα1-6)Manβ1-4GlcNAcβ1-4GlcNAcβ-LVaNKT |
| 402 | Galα1-3Galβ1-4GlcNAcβ1-3GalNAcα-Sp14 |
| 403 | Galβ1-3GlcNAcβ1-6Galβ1-4GlcNAcβ-Sp0 |
| 404 | Galβ1-3GlcNAcα1-6Galβ1-4GlcNAcβ-Sp0 |
| 405 | GalNAcβ1-3Galα1-6Galβ1-4Glcβ-Sp8 |
| 406 | GlcNAcβ1-6(GlcNAcβ1-3)GalNAcα-Sp14 |
